# Gene expression profile of rat left ventricles reveals persisting changes following chronic mild exercise protocol: implications for cardioprotection

**DOI:** 10.1186/1471-2164-10-342

**Published:** 2009-07-30

**Authors:** Betti Giusti, Marina Marini, Luciana Rossi, Ilaria Lapini, Alberto Magi, Andrea Capalbo, Rosa Lapalombella, Simona di Tullio, Michele Samaja, Fabio Esposito, Vittoria Margonato, Maria Boddi, Rosanna Abbate, Arsenio Veicsteinas

**Affiliations:** 1 Department of Medical and Surgical Critical Care and Center of Research, Transfer and High Education, "DENOTHE", University of Florence, Florence, Italy; 2 Department of Histology, Embryology and Applied Biology, University of Bologna, Bologna, Italy; 3 S. Maria agli Ulivi, Don C. Gnocchi Foundation, Florence, Italy; 4 Department of Medicine, Surgery and Dentistry, University of Milan, San Paolo Hospital, Milan, Italy; 5 Department of Sport Sciences, Nutrition and Health, University of Milan, Milan, Italy; 6 Center of Sport Medicine, Don C. Gnocchi Foundation, Milan, Italy

## Abstract

**Background:**

Epidemiological studies showed that physical exercise, specifically moderate lifelong training, is protective against cardiovascular morbidity and mortality. Most experimental work has focused into the effects and molecular mechanisms underlying intense, rather than mild exercise, by exploring the acute effect of training. Our study aims at investigating the cardioprotective effect of mild chronic exercise training and the gene expression profile changes at 48 hrs after the exercise cessation. Rats were trained at mild intensity on a treadmill: 25 m/min, 10%incline, 1 h/day, 3 days/week, 10 weeks; about 60% of the maximum aerobic power. By Affymetrix technology, we investigated the gene expression profile induced by exercise training in the left ventricle (LV) of trained (n = 10) and control (n = 10) rats. Cardioprotection was investigated by ischemia/reperfusion experiments (n = 10 trained vs. n = 10 control rats).

**Results:**

Mild exercise did not induce cardiac hypertrophy and was cardioprotective as demonstrated by the decreased infarct size (p = 0.02) after ischemia/reperfusion experiments in trained with respect to control rats. Ten genes and 2 gene sets (two pathways) resulted altered in LV of exercised animals with respect to controls. We validated by real-time PCR the increased expression of four genes: similar to C11orf17 protein (RGD1306959), caveolin 3, enolase 3, and hypoxia inducible factor 1 alpha. Moreover, caveolin 3 protein levels were higher in exercised than control rats by immunohistochemistry and Western Blot analysis. Interestingly, the predicted gene similar to C11orf17 protein (RGD1306959) was significantly increased by exercise. This gene has a high homology with the human C11orf17 (alias: protein kinase-A interacting protein 1 or breast cancer associated gene 3). This is the first evidence that this gene is involved in the response to the exercise training.

**Conclusion:**

Our data indicated that few, but significant, genes characterize the gene expression profile of the rat LV, when examined 48 hrs since the last training section and that mild exercise training determines cardioprotection without the induction of hypertrophy.

## Background

A large number of epidemiologic studies have emphasized the relationship between increased physical activity during leisure time to both the reduced progression of atherosclerosis [1] and risk of cardiovascular disease [2,3]. Exercise has been related to reduced post-ischemia infarct size, in a manner dependent on the regularity and intensity of exercise [4-8]. Recent studies have clearly shown that training at moderate to high intensity increases myocardial tolerance to ischemia/reperfusion (I/R) [9], improves cardiac performance and cell defence capacity against stress [10,11]. It has been suggested that the mechanisms underlying training-induced cardioprotection resemble those evidenced by ischemic preconditioning, e.g. the phenomenon whereby short ischemic episodes given before a major ischemic insult lead to endogenous cardioprotection [12]. Ischemic preconditioning results in cardioprotection within two time windows: a first window within 2–3 hours from the injury and a second window 24–72 hours later [13].

Recently, mild chronic exercise training (14 weeks, increasing in intensity to 55% of the maximum aerobic power, VO2max), which resembles the procedure recommended for middle-aged human beings (Recommendations of the American College of Sport Medicine, http://www.acsm.org//AM/Template.cfm?Section=Home_Page), was demonstrated to protect the heart against I/R [14] in a similar way to more intense training programs [9-11]. The cellular and molecular mechanisms by which exercise training, and in particular mild exercise training, exerts its effects on the heart are partially understood due, at least in part, to the complex and pleiotropic nature of the exercise stimulus. A possible way to approach this fundamental issue is to use microarray technology to identify differentially expressed transcripts. In fact, whole genome technologies may facilitate the identification of genes or gene sets/pathways that have not so far been associated with exercise training. Reports based on the microarray approach are beginning to emerge concerning the evaluation of responses to the physical exercise of leukocytes [15], skeletal muscle [16], and left ventricle (LV) [9,17,18].

In order to get a greater insight into the molecular mechanisms underlying the beneficial effects of mild physical exercise, we investigated the cardioprotective effect and evaluated gene expression changes induced by mild chronic exercise training in the LV of the rat. We focused into the gene expression changes at 48 hours after the last exercise session for the purpose of: a) characterizing the "permanent" instead of acute gene expression profile in response to physical exercise, and b) comparing and extending previously obtained data by including larger gene sets and population [14].

## Methods

### Animals and experimental design

Animal handling, training protocol and mode of sacrifice were approved by the Ethical Committee on the Use of Laboratory Animals of the Health Authority of Milan (Italy) according to the 86/609/CEE guidelines. Training was carried out according to the American Physiological Society guidelines for exercising rodents on treadmills (American Physiological Society, 2006 http://www.the-aps.org/pa/action/exercise/book.pdf). The investigation conforms to the Guide for the Care and Use of Laboratory Animals published by the National Institutes of Health [19]. We used 40 male Sprague-Dawley-CD rats (8 weeks old, 360 ± 15 g body weight at the beginning of the study), randomly divided into two groups: exercise-trained (TRA) animals and sedentary controls (CTR). During the experimental period, all animals had free access to water and a conventional laboratory diet (Standard diet n.48, Laboratorio Dr Piccioni, Milan, Italy) until sacrifice. Room temperature was kept at 21 ± 2°C and 12 h of light was automatically alternated to 12 h of darkness. Rats were periodically examined by a veterinarian. Food consumption and body weight were evaluated three times a week. Their internal organs, as examined by a pathologist on the day of sacrifice, appeared to be normal and disease free.

### Exercise training

After one week of acclimatization, 20 animals were randomly chosen to run on a treadmill (Medical Instruments, U.S.A.) at 10%-grade. The training protocol required two weeks of gradually increasing effort and time of exercise up to 25 m/min for 1 h/day, 3 days/week, for a total of 10 weeks. According to previous studies [20,21], the training exercise intensity used in the present work corresponded to ~60% of the maximum aerobic power VO_2max _[20]. Control animals (n = 20) were placed on a non-moving treadmill during the training sections.

Forty eight hours after the last run, 10 TRA rats and 10 CTR rats were anesthetized (100 mg/kg ip heparinized sodium thiopental), sacrificed, quickly dissected, and then heart and soleus muscles were removed. Blood from the heart was accurately squeezed out and soleus muscles were blotted and freed of connective tissue before they were weighed.

Left ventricles were immediately frozen in liquid nitrogen and stored at -80°C until RNA and protein extraction for microarray, real time (RT)-PCR, western blot analyses and immunohistochemistry assay were performed.

### Total RNA extraction and gene expression profiling by Affymetrix GeneChip analysis

Frozen cardiac tissue was reduced to powder by means of a sterilized ceramic mortar and pestle. RNA was isolated by using TRIZOL™ (Invitrogen, Milan, Italy), as suggested by the manufacturer. The presence of RNA degradation was assessed by evaluation of 28S and 18S band sharpness after denaturing electrophoresis. Quantification and purity of RNA samples was performed by 260 and 280 spectrophotometer analysis; a 260/280 absorbance ratio ranging between 1.8 and 2.1 being acceptable. We determined, by microarray technology, the contemporary expression of 31,000 probe sets (each set of 25-mer oligonucleotides on the GeneChip recognized a transcript) representing 30,000 transcripts and variants from over 28,000 well-substantiated rat genes. To this purpose we used the Affymetrix GeneChip technology and GeneChip Rat Genome 230 v2.0 Array (Affymetrix, Santa Clara, CA). Standard protocols for chip hybridizations (available at http://www.affymetrix.com), One-Cycle Target Labeling kit, and Control Reagents kit were used (Affymetrix, Santa Clara, CA). Briefly, double-stranded cDNA was synthesized by reverse transcriptase reaction from total RNA extracted from the LV of all animals (CTR and TRA). cDNA was then used to obtain biotin-labeled cRNA by in vitro transcription reaction. After the clean-up procedure of biotinylated cRNA, the cRNA was fragmented before hybridization. After an over-night hybridization, GeneChips were washed and stained with streptoavidin-phycoerythrin conjugate and biotinylated anti-streptavidin antibody. RNA from individual animals was hybridized to each chip. After the scanning procedure, data files were checked for quality parameters (such as scale factor, background noise, corner and central fluorescent intensities, GAPDH and Actin 3'/5' ratio values and percentage of expressed probe sets). Microarray data analysis was performed according to Affymetrix suggestions (GeneChip Expression Analysis: data analysis fundamentals in http://www.affymetrix.com) and was based on our previous experience in microarray technology [22,23].

### Data Extraction and Statistical Analysis

Data reported in this publication have been deposited in the National Center for Biotechnology Information (NCBI) Gene Expression Omnibus (GEO; http://www.ncbi.nlm.nih.gov/geo) and are accessible through GEO Series accession number GSE7640 (to allow review of GSE7640 before the manuscript acceptance: http://www.ncbi.nlm.nih.gov/geo/query/acc.cgi?token=jpeltsoyskkoklw&acc=GSE7640).

Image and expression data files were generated with Affymetrix MAS 5.0. The average percentage of probe sets with "present calls" (qualitative detection of transcripts) was 48.1%, indicating that the array hybridizations were of good quality. Low level and statistical analysis was done using R 2.3. Microarray data were first processed in R environment http://www.r-project.org by Affymetrix package to identify present/absent probe set, and then subjected to a normalization step. We normalized data according to the MicroArray Suite (MAS) method: background correction with MAS method, normalization at a probe level with constant method (a global adjustment by a constant value to equalize the chip-wide mean signal intensity between chips) [24], and summary of the probe set intensities with the method of averaged differences http://www.affymetrix.com, http://www.bioconductor.org.

In order to identify differentially expressed genes, at statistically significant level, in LV of TRA and CTR rats, we applied a t-statistic variant approach. We used the significance analysis of microarrays (SAM) method [25], in which the t-statistic has a constant value added to the standard deviation. We performed SAM analysis with Siggenes package http://www.bioconductor.org[26]. The minimization of the FDR allowed us to identify 12 differentially expressed probe sets with a delta equal to 0.7 and FDR 19%. All the analysis was written in the freely available statistical language R.

In order to increase the information content of our dataset, Gene Set Analysis (GSA) was applied using the GSA R package http://www-stat.stanford.edu/~tibs/GSA/. GSA is a computational method that determines whether an a priori defined set of genes shows statistically significant concordant differences between two biological states. This method allowed us to investigate whether genes involved in the same biological functions or pathways resulted co-regulated, even if they did not result differentially expressed at statistically significant levels by SAM method. GSA is a modification of the Gene Set Enrichment Analysis (GSEA) [27] that makes use of the "maxmean" statistic instead of Kolmogorov-Smirnov statistic to score gene sets. The analysis was performed on 459 a priori defined gene sets (a gene set is defines by genes involved in the same biological process, molecular function, cellular component, or pathway) that were downloaded at the Molecular Signatures Database site http://www.broad.mit.edu/gsea/msigdb/index.jsp.

### Validation by Real-Time PCR

Expression patterns of 4 genes [similar to C11orf17 protein (RGD1306959), caveolin 3 (Cav3), enolase 3 (Eno3) and hypoxia inducible factor 1 (Hif-1)] were validated by RT-PCR. Reverse transcription was performed making use of Omniscript Reverse Transcription Kit (Qiagen, Hilden, Germany). The primer sequences, the size of the amplification products and the mean efficiency of the amplification reactions are given in Additional File 1. Primers were chosen by using the *AMPLIFY *free software; whenever possible, primers were designed to span an exon-exon junctions and were localized at the 3' end; for similar to C11orf17 protein (RGD1306959) gene, the primers were designed away from the 3' end because of the similarity of its 3' sequence with that of other genes, as examined by nBLAST analysis. Primers were obtained from PROLIGO (Proligo, France SAS). The presence of unspecific products was ruled out after analyzing the melting curve of the amplicons. RT-PCR was performed in an ABI PRISM 5700 real-time thermal cycler using the SYBR Green kit (Qiagen). The reactions were performed in quadruplicate; the final volume (25 μL) contained QuantiTect SYBR Green PCR Master Mix, primers (0.3 μM each), and 10 ng of cDNA. Inter-plate differences were less than 3.5% and intra-plate differences were even lower. Standardization curves were run in the range from 2.5 to 40 ng cDNA input and were highly linear (Pearson correlation coefficient *r *> 0.997). PCR efficiencies were calculated from the slopes obtained by ABI PRISM software; the efficiency (*E*) of each PCR cycle in the exponential phase was calculated according to the equation *E = 10(-1/slope)*. For each gene, differences in the PCR efficiency of the 20 samples were less than 5% and were not considered in the final calculations. The relative gene quantification method developed by Pfaffl [28] was applied, one of the samples having been randomly chosen as a control. Expression of target genes was calculated based on *E *and the crossing point deviation of unknown samples versus a control and expressed in comparison to the housekeeping gene. Values are then expressed in arbitrary units, setting to 100 the amount of RPL13a in 10 ng cDNA input.

### Western Blot

LV cryomicrotome slices were lysed in RIPA buffer. Lysates (30 μg) were run in SDS-PAGE minigels (12% acrylamide-bis acrylamide, 30:1) and blotted into nitrocellulose membranes by standard procedures. Rabbit anti-rat monoclonal antibodies for caveolin-3 immunodetection were obtained from BD Transduction Lab, Lexington, while mouse anti-rat beta-actin polyclonal antibodies were obtained from Sigma and were used at 1:2000 and 1:200 dilution, respectively. HRP-conjugated secondary antibodies (1:5000) were purchased from Sigma. HRP chemiluminescent reaction was developed using Super Signal West Dura Extended Duration (Pierce, Rockford, IL) and used to expose Kodak Biomax Light film. The spots were quantified by means of BioRad GelDoc 2000 and results were expressed as caveolin-3 to beta-actin ratio.

### Immunohistochemistry

For the quantification of caveolin-3, LV sections (5 μm-thick) were obtained from the frozen specimens with random orientation; they were fixed in cold 4% paraformaldehyde, marked with mouse anti-rat monoclonal antibodies for caveolin-3 (diluted 1:100, BD Transduction Lab, Lexington), followed by incubation with Super Sensitive Polymer-HRP IHC Biogenex, USA and development of the chromogen reaction with 3,3'diaminobenzidine (DAB). The slides were assigned conventional numbers, so that the operator was blind to the training status of the rat. Under the light microscope, at 250× magnification, 30 non-overlapping fields were randomly selected for each section, avoiding areas with holes, folds, staining defects or large vessels. Images were acquired by a 3 CCD (charge-coupled device) color video camera (KY F55B, JVC Italia, Milan, Italy), and examined with an image processing software (Image-Pro Plus, Media Cybernetics, Silver Spring, MD, USA). A specific function of the software allows the operator to interactively define the threshold for the automatic measurement of the structures intensely brown-stained, excluding any other structure less intensely stained in brown (background staining) or stained with other colors. By means of this system, for each microscopic field evaluated, the area stained with anti-caveolin-3 antibody, identified by an intense brown staining, was measured and expressed as a percentage of the total area. The total surface thus evaluated per condition was 2.7 mm^2^. Some slides were counterstained with hematoxylin, in order to appreciate the tissue morphology.

### Immunofluorescence labeling

In order to identify caveolin-3 (+) cells, heart cryo-sections were double labeled with a) mouse anti-rat monoclonal antibodies for caveolin-3 (diluted 1:100, BD Transduction Lab, Lexington), plus FITC-conjugated rabbit anti-mouse polyclonal antibody (diluted 1:250, DAKO, Germany) and b) goat anti-rat polyclonal antibody for myosin heavy chain (diluted 1:100, Santa Cruz Biotechnology Inc., Santa Cruz, Ca) plus TRITC-conjugated bovine anti-goat polyclonal antibody (diluted 1:100, Santa Cruz Biotechnology Inc., Santa Cruz, Ca). Slides were mounted with Pro Long Antifade Reagent with DAPI (Molecular Probes, Canada).

### Ischemia reperfusion experiments

Forty eight hours after the last training session, 10 exercised rats and 10 control rats were used for I/R experiments as previously described [14]. Briefly, rats were anesthetized by intra-peritoneal injection of sodium pentobarbital (50 mg/kg). Body temperature was kept constant at 37°C by use of a heating platform controlled by a thermostat. Animals were intubated through a tracheotomy and ventilated mechanically (tidal volume 3 mL; ventilation rate 50 strokes/min). A left thoracotomy was performed between the 3^rd ^and 4^th ^rib to allow access to the heart. A silk suture (6/0) was passed around the left coronary artery and a small polyethylene catheter was used to form a snare. All rats were allowed 10 min after the completion of the surgical preparation to reach a steady state before beginning the protocol. The left coronary artery branch was occluded by pulling the snare and the occluded position was maintained for 30 min by means of a haemostatic clamp. Hearts were reperfused for 90 min by releasing the snare, and then they were removed, cannulated via the aorta and perfused with 15–20 mL saline at room temperature to wash out the blood. The left coronary branch was re-occluded and a saturated Evans blue solution (2 mL) was injected through the aorta and upstream the occlusion to mark the ischemic zone as the area without the dye. Hearts were then briefly frozen in liquid nitrogen and stored at -20°C.

To measure the infarcted area, hearts were cut into five or six transverse slices (1 mm thick). Slices were incubated in triphenyltetrazolium chloride in sodium phosphate buffer at 37°C for 20 min to stain viable cells in the risk zone. Finally, the slices were immersed in 10% formalin for 4 days to enhance contrast between stained and unstained areas. Stained and unstained areas were calculated from computerized images of the slices using NIH Image software (NIH AutoExtractor 1.51; National Institutes of Health) and averaged for all the slices.

### Statistical Analysis

The data were analyzed by using SPSS (version 11.5). All values were expressed as median and range unless indicated differently. When comparing groups, statistical significance was determined by using non-parametric Mann-Whitney test. A p value less than 0.05 was considered statistically significant.

## Results

### Animal characteristics

The physical characteristics of the animals at the end of the training period are reported in Table 1. Although TRA rats were slightly leaner than CTR rats (p = ns), no statistically significant differences in heart weight, in heart-to-body weight ratio or in the thickness of the left ventricular wall were observed. This indicates that training did not induce cardiac hypertrophy. However, two evidences showed that animals had been efficiently trained: i) the soleus to body weight ratio was higher in TRA than in CTR rats; and, moreover, ii) during the second and the last week of training each rat was evaluated for maximal endurance running capacity, which resulted to be in the 15–25 min range while running at 15 m/min in the second week and in 100–120 min range at 25 m/min in the last week.

**Table 1:** Animal characteristics.

	Control rats	Exercise-trained rats	p
**Number of animals**	10	10	
**Body weight, g**	588 (496–629)	567 (519–613)	ns
**Heart weight, g**	1.41 (1.30–1.56)	1.40 (1.23–1.60)	ns
**Heart/body weight ratio**	2.46 (2.27–2.66)	2.48 (2.34–2.80)	ns
**Left ventricle wall thickness, mm**	4.16 (3.70–5.10)	4.08 (3.55–5.02)	ns
**LV/(RV+S)**	0.95 (0.81–1.04)	0.91 (0.84–1.47)	ns
**Soleus/body weight ratio**	0.38 (0.30–0.48)	0.45 (0.40–0.51)	0.002

LV/(RV+S) = Left ventricle/(right ventricle + septum) wall thickness. Data are expressed as median and range.

### Affymetrix Gene Expression Profiling of exercise-trained and control rats

After data processing and application of the filtering criteria, the average of analyzable probe sets numbered 14,911. The full list of the 300 most expressed genes in the overall animal population (n = 10 CTR + n = 10 TRA), as well as the Gene Ontology analysis (for biological process and molecular function) are available as Additional File 2, 3 and 4. The 300 most expressed genes in LV are involved in the following biological processes: protein biosynthesis, electron transport, muscle development, regulation of heart contraction, fatty acid metabolism, ATP synthesis coupled proton transport, glycolysis, response to oxidative stress, cytoskeleton organization and biogenesis, protein folding, signal transduction and protein modification. By using the SAM method to assess the expression of the 14,911 called transcripts, we observed 12 transcripts (10 genes) differentially expressed in LV hearts of TRA animals with respect to CTR controls (Table 2). All differentially expressed genes showed an increased expression in TRA animals with respect to CTR controls.

**Table 2:** Differentially expressed genes between control (n = 10) and trained (n = 10) rat groups.

Gene Name (Gene Symbol)	Gene ID	GenBank	Probe name	D	r
similar to C11orf17 protein (RGD1306959)	361624	BF561368	1392938_s_at	5.039	1.5

caveolin 3 (Cav3)	29161	NM_019155	1387814_at	4.216	1.4

similar to RIKEN cDNA 1700012G19 gene (RGD1307773)	287115	BG380656	1388881_at	4.206	1.2

similar to C11orf17 protein (RGD1306959)	361624	AA799992	1385458_a_at	4.168	1.6

enolase 3, beta (Eno3)	25438	NM_012949	1386907_at	4.135	1.5

similar to C11orf17 protein (RGD1306959)	361624	BF561368	1383175_a_at	3.947	1.5

cytochrome P450, family 27, subfamily a, polypeptide 1 (Cyp27a1)	301517	M73231	1387914_at	3.927	1.4

similar to RIKEN cDNA 2700002I20 (RGD1307279)	307210	AI171367	1373074_at	3.783	1.3

EGL nine homolog 1 (Egln1)	308913	BI282122	1389207_at	3.652	1.2

Unknown	NA	BI295165	1373167_at	3.594	1.2

cystatin C (Cst3)	25307	BG666933	1370855_at	3.457	1.3

tumor necrosis factor, alpha-induced protein 1 (endothelial) (Tnfaip1)	287543	BM390023	1371911_at	3.433	1.2

Probe name = Affymetrix number of the probe set that recognized the specific transcript; d = significance analysis of microarrays (SAM) t-statistic; r = fold change according to SAM [mean(TRA intensities)/mean(CTR intensities)].

We focused our attention into three genes that microarray analysis placed on top of the list on the differentially regulated genes [similar to C11orf17 protein (RGD1306959), Cav3 and Eno3], and one gene (Hif-1alpha), which was not found different between TRA and CTR rats by the microarray analysis. We tested Hif-1 alpha gene by a further method with a different sensitivity, RT-PCR, because it may be potentially involved in the modulation of the expression of two up-regulated genes [Eno3 and EGL nine homolog 1 (Egln1)]. Interestingly, one predicted gene, similar to C11orf17 protein (RGD1306959), was found to be significantly increased by three different probe sets, suggesting the potential role of this gene in the cardioprotective effect of exercise (Table 2).

### GSA analysis

In order to identify functional gene sets correlated with the mild chronic exercise training, we applied GSA analysis. GSA analysis identified 2 gene sets out of 459 whose expression was correlated with exercise training, namely ARENRF2PATHWAY (http://www.biocarta.com/pathfiles/h_ARENRF2PATHWAY.asp; p < 0.0001 and FDR = 0) and GABAPATHWAY (http://www.biocarta.com/pathfiles/h_GABAPATHWAY.asp; p < 0.0001 and FDR = 0) (Table 3).

**Table 3:** Genes in the ARENRF2PATHWAY (Antioxidant Response Elements Nrf1 and Nrf2 pathway) and GABAPATHWAY (Gamma-AminoButyric Acid pathway).

GENE NAME	GENE SYMBOL
**ARENRF2PATHWAY** http://www.biocarta.com/pathfiles/h_ARENRF2PATHWAY.asp	

cAMP responsive element binding protein 1	Creb1
FBJ murine osteosarcoma viral oncogene homolog	Fos
FXYD domain-containing ion transport regulator 2	Fxyd2
Jun oncogene	Jun
Kelch-like ECH-associated protein 1	Keap1
mitogen activated protein kinase 1	Mapk1
mitogen activated protein kinase 14	Mapk14
nuclear factor, erythroid derived 2, like 2	Nfe2l2
protein kinase C, alpha	Prkca
protein kinase C, beta 1	Prkcb1
v-maf musculoaponeurotic fibrosarcoma oncogene family, protein G	Mafg
v-maf musculoaponeurotic fibrosarcoma oncogene family, protein K	Mafk

**GABAPATHWAY** http://www.biocarta.com/pathfiles/h_GABAPATHWAY.asp	

dynamin 1	Dnm1
gamma-aminobutyric acid (GABA-A) receptor, subunit alpha 1	Gabra1
gamma-aminobutyric acid (GABA-A) receptor, subunit alpha 3	Gabra3
gamma-aminobutyric acid (GABA-A) receptor, subunit alpha 4	Gabra4
gamma-aminobutyric acid (GABA-A) receptor, subunit alpha 5	Gabra5
gamma-aminobutyric acid (GABA-A) receptor, subunit alpha 6	Gabra6
gamma-aminobutyric acid receptor associated protein	Gabarap
Gephyrin	Gphn
N-ethylmaleimide sensitive fusion protein	Nsf
Rous sarcoma oncogene	Src
ubiquilin 1	Ubqln1

The ARENRF2PATHWAY (Oxidative Stress Induced Gene Expression Via Nrf2) is a pathway involved in the oxidative stress through Nrf1 and Nrf2 transcription factors that bind to antioxidant response elements (AREs) (Table 3). The GABAPATHWAY (Gamma-aminobutyric Acid Receptor Life Cycle) is a pathway that includes genes involved in the gamma-aminobutyric acid signaling responsible for important cellular functions such as synaptic transmission, ion channel activity, and oxidative stress (Table 3).

### Validation analysis by RT-PCR of RGD1306959, Cav3, Eno3 and Hif1a gene expression

**Figure 1:**
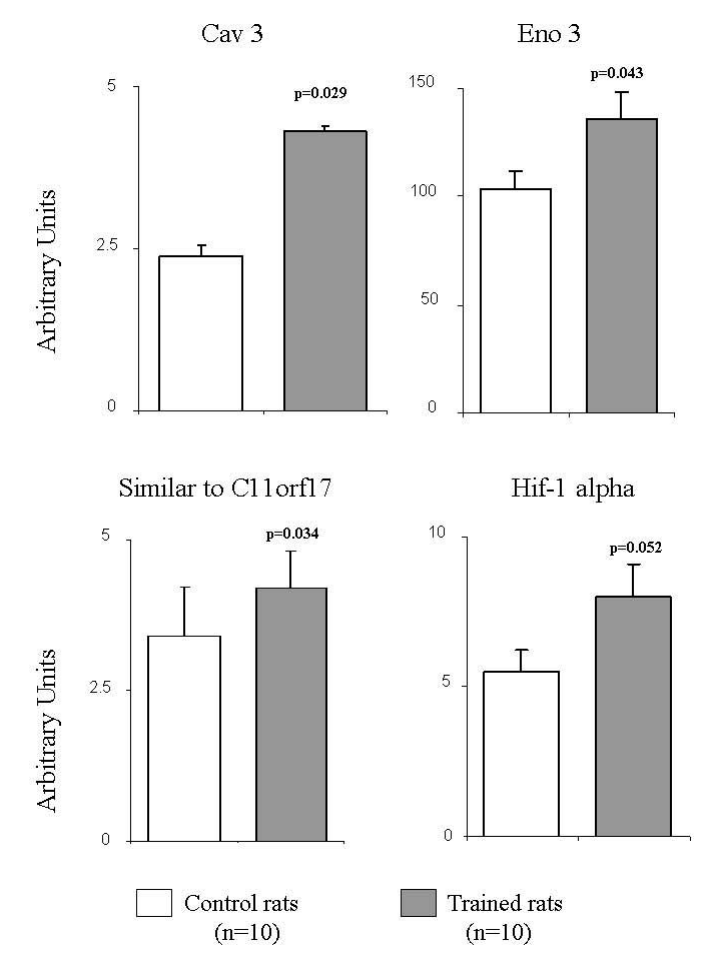
**Gene expression analysis by real time RT-PCR of similar to C11orf17 protein (RGD1306959), caveolin 3, enolase 3 and Hif-1 alpha genes in trained and control rats**. Data are expressed as mean ± SE; p-values are referred to trained vs. control rats.

RT-PCR analysis confirmed the increased expression, previously observed by GeneChip Affymetrix technology, of Cav3, Eno3 and RGD1306959 genes in moderately trained rats with respect to control rats (p = 0.029, p = 0.043, and p = 0.034 respectively) (Figure 1). As concerns Hif-1 alpha gene expression, the microarray data were actually confirmed by RT-PCR, since it narrowly failed to reach the statistical significance (p = 0.052) between CTR and TRA rats.

### Western Blot and Immunohistochemistry analysis of caveolin 3

**Figure 2:**
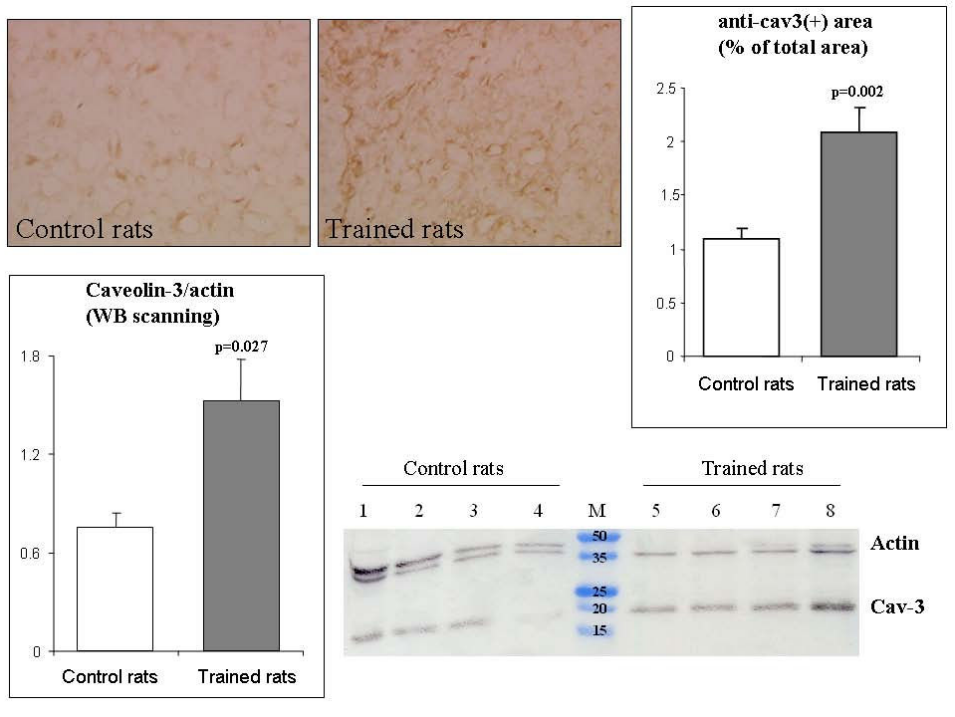
**Immunohistochemistry and western blot quantification of caveolin-3**. Top: Left ventricle slides were obtained from frozen tissue by a cryotome and stained with anti caveolin-3 antibody, followed by HRP-conjugated secondary antibody and DAB reaction. One representative picture of cardiac tissue from controls and trained rats is shown. Immunohistochemistry images were analyzed by a software, which allowed the evaluation of the percentage of the area that reacted with anti caveolin-3 antibody. P-values are referred to trained vs. control rats. Bottom: Representative Western Blot assay with anti caveolin-3 antibodies. Bands of approximately 18 kDa correspond to caveolin 3 monomer. Cytoplasmic actin (43 kDa) was immunostained for equal loading control in the same gel. Lanes 1 to 4: protein extracted from left ventricle of control rats. M: markers. Lanes 5 to 8: protein extracted from left ventricle of exercise trained rats. The Western Blot was scanned by a densitometer and caveolin-3/actin ratio was found significantly larger in blots from trained rat hearts respect to controls.

As analyzed by Western Blot, the protein level of caveolin 3 in left ventricles of TRA rats was significantly higher with respect to CTR rats (p = 0.027) in agreement with the mRNA expression level (Figure 2).

Both sedentary control rats and exercise trained rats resulted positive for immuno-staining of the caveolin 3 (Figure 2). A stronger staining (p = 0.0024) was observed in exercise trained animals with respect to sedentary controls (Figure 2).

**Figure 3:**
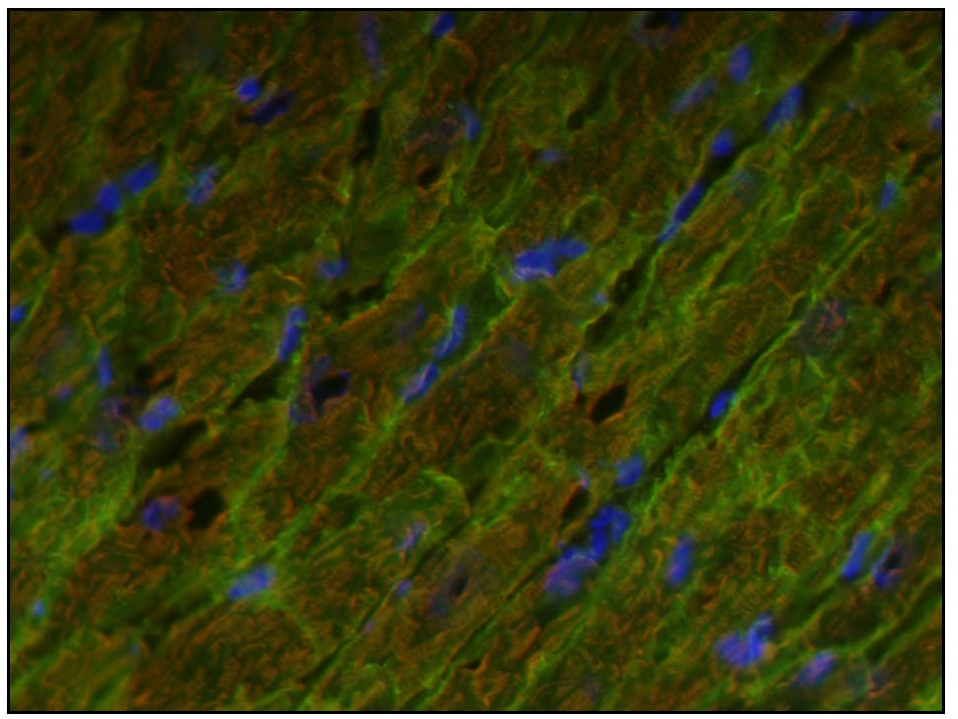
**Fluorescence microscopy view of a longitudinal section of cardiomyocytes from a trained rat heart, double labeled with anti-caveolin 3 antibody (green) – staining the cell membranes – and anti-myosin heavy chain (red) – staining the sarcomers**. Nuclei have been counter labeled in blue. Magnification: 400×.

Double labeling of cryo-sections from trained rat hearts with anti-caveolin 3 and anti-myosin heavy chain antibodies showed that the cells displaying intense membrane positivity for caveolin 3 also stained for sarcomeric myosin, thus indicating that caveolin 3 is increased in cardiomyocytes (Figure 3).

### Ischemia-reperfusion

**Figure 4:**
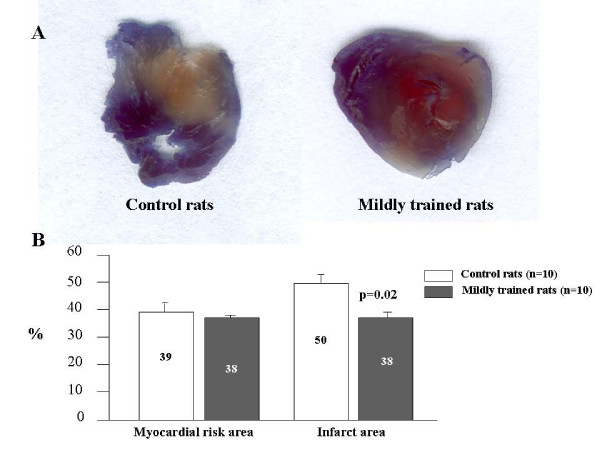
**I/R experiments: A = images of a heart from control and mildly exercised rats: blue = perfused myocardium; red = area at risk; white = infarct tissue**. B = mean percentages ± SE of myocardial risk areas at the end of 30 min-ischemia followed by 90 min-reperfusion and of infarct areas (values are representative of total ventricle percentage).

In order to evaluate the cardioprotective effect of the mild chronic exercise training protocol, I/R experiments were performed. Figure 4 summarizes the I/R findings by reporting a slice of the myocardium from a control (left) and a trained (right) animal. The red and blue areas represent, respectively, the area at risk of infarct and the perfused area. The mean risk area, expressed as a percentage of total ventricle area, i.e., the sum unstained plus red-stained area with respect to total ventricle area, was 39.0% (± 2.1%) and 38.0% (± 0.8%) in control and trained rats (p = ns). As these data were essentially the same in the two groups, one can derive that the severity of left anterior descending artery (LAD) occlusion was the same. By contrast, the white area, that represents the infarct size, when expressed as a percentage of the risk area, was larger in control than in trained animals [50.0% (± 3.9%) vs. 37.0% (± 1.1%), p = 0.02], indicating improved myocardial protection in trained animals.

## Discussion

In this study, we show that the 10-week mild exercise aerobic training (~60% VO2max) induces cardioprotection to I/R and alters the gene expression profile in rat left ventricles at 48 hours after the last training session. The differentially expressed genes were: Cav3; Eno3; cytochrome P450, family 27, subfamily a, polypeptide 1 (Cyp27a1); Egln1; cystatin C (Cst3) and tumor necrosis factor, alpha-induced protein 1 (endothelial) (Tnfaip1) genes, along with the two RGD1306959 and RGD1307773 predicted genes and two unidentified clones. Present data indicate that the applied mild chronic training protocol is effective, as demonstrated by the higher running capacity and the soleus to body weight ratio in TRA rats with respect to CTR rats, without inducing cardiac hypertrophy, as demonstrated by the absence of differences in heart-to-body weight ratio and in the thickness of the left ventricular wall between TRA and CTR rats.

The described mild exercise training model determined development of cardioprotection, as evaluated by I/R experiments, similarly to what reported in the case of intense training procedures [29], as demonstrated by the fact that the infarct size was larger in CTR than in TRA. These data are coherent with the previous observation obtained by our group with a similar training protocol [14].

The selected experimental design satisfies several purposes. First, the applied mild training protocol resembles that practiced by adult humans for cardiovascular fitness. Ten-week training duration enabled the onset of cardiac adaptations and allowed for the estimation of the possible occurrence of cardiac hypertrophy [9,17,18]. Second, rats were sacrificed 48 hours after the last training session in order to rule out acute effects of exercise and to evaluate gene expression changes due to chronic adaptation. It might be hypothesized that the cardioprotection resulting from the adaptation to mild chronic exercise training is the result of a summation of several second windows of protection similar to what observed during ischemic preconditioning. In fact, transient ischemic events may occur during exercise at high heart rate, when the time available for diastolic coronary flow is reduced and myocardial contractility as well as mechanical compression is likely to impede coronary flow [30]. Moreover, the experimental design allowed us to compare and extend on a larger number of genes data reported in a previous paper [14] employing a similar chronic mild training protocol.

Gene expression profile changes induced by exercise training have been investigated in previous studies that employed training procedures of a more intense nature than those used in this study and that, in contrast to the protocol of this study, induced cardiac hypertrophy [9,17,18]. Differentially expressed genes reported in those studies differ highly in number and type, not only when compared to those reported in the present work, but also when a comparison is drawn among them. Conflicting results may stem from the intensity, duration of protocols, and distance of the sacrifice from the last exercise bout. However, it is interesting to note that in the studies cited above the number of differentially expressed genes decreased according to the time that elapsed from the last exercise bout to the sacrifice, suggesting a restoring of gene expression to levels comparable to those of sedentary control animals: 24 hours (305 genes) [9], 48 hours (75 genes) [17], 72 hours (27 genes) [18].

Recently, Freimann and coworkers [11,31] evaluated gene expression profiles of heart from rats which received a 7-week swimming exercise training and underwent surgically-induced myocardial infarction. The gene expression profiles were evaluated before, 4 hours, 2 days and 4 weeks after surgery. Differentially expressed genes did not overlap, at any time point, with those reported here. However, the experimental design of the Freimann's group differed from ours as we evaluated the gene expression profiles that characterized animals after the mild chronic exercise and, in a different set of rats trained in parallel using an identical training protocol, we determined the cardioprotection by measuring the extent of the injury caused by ischemia/reperfusion experiments. Concerning the previous paper of our group [14], none of the 14 differentially expressed genes identified (96 candidate genes involved in stress and toxicity) was included within the genes found differentially expressed in the present study. On the other hand, two different technologies – a macroarray focused on 96 genes in the previous paper and Affymetrix GeneChips representing 31.000 transcripts in the present paper – were used.

Among the differentially expressed transcripts, we pointed our attention on 3 genes: similar to C11orf17 protein (RGD1306959), Eno3 and Cav3.

The predicted sequence of similar to C11orf17 protein (RGD1306959) has a high homology (mRNA nucleotide: 90%–92%; protein: 77% on positivity) with the human C11orf17 [alias: protein kinase-A interacting protein 1 (AKIP1) or breast cancer associated gene 3 (BCA3)]. C11orf17 has been recently shown to enhance NF-κB-mediated gene expression [32], a redox-sensitive transcription factor, known to be involved in the control of a large number of cellular processes, such as immune and inflammatory responses, developmental processes, cellular growth, and apoptosis. The gene expression of similar to C11orf17 protein (RGD1306959) gene was found increased in LV of hypertensive rats and positively correlated with LV mass index, thus pointing to its potential implication in LV hypertrophy development [33]. Interestingly, in these hypertensive rats, Eno3 showed a negative correlation with LV mass index [33], whereas, in our model of mild physical exercise without cardiac hypertrophy, Eno3 gene showed an increased expression in trained rats. Our findings, together with data obtained in hypertensive rat models, strongly suggest a relevant role of similar to C11orf17 protein (RGD1306959) gene in the adaptive response of the cardiac muscle mass to the workload; this mechanism could be balanced by the up-regulation of the Eno3 gene. Eno3 gene encodes one of the three enolase isoenzymes found in mammals. This isoenzyme is found in skeletal and cardiac muscle cells. The functional role of this gene in heart muscle, as well as skeletal muscles, has not been elucidated yet. Several reports described a role for the hypoxia-inducible factor-1 (HIF-1) in the transcriptional activation of enolase-1 [34], and enolase-2 genes [35]. In the present study, Hif-1alpha showed a trend toward increased expression in trained rats, thus suggesting that Hif-1alpha could be regulated also by non-hypoxic stimuli. In a recent proteomics study, enolase 1 alpha and proteins similar to alpha enolase have been found to increase in exercised rats [36]. Moreover, beta enolase was observed to increase in athletes after running a race [37]. These data further indicate an important role of beta enolase in the preconditioning phenomena that induce cardioprotection.

Using the present protocol of mild exercise training an increased expression of caveolin 3 at transcriptomic and proteomic levels was demonstrated. In cardiomyocytes, caveolin 3 is a major constituent of caveolae, plasma membrane invaginations implicated in vesicular trafficking, signal transduction, and Ca^2+ ^homeostasis [38]. The influence of caveolin 3 on hypertrophy has been previously suggested by a number of evidences, including the development of cardiac hypertrophy in caveolin 3-null mice [39], the inhibition of agonist-induced cardiomyocyte hypertrophy by overexpression of caveolin 3 [40], and the observation that exercise training, preventing pathological hypertrophy in spontaneously hypertensive rats, is associated to the overexpression of caveolin 3 [41]. Moreover, it has been found that caveolin 3 induces protection of cardiac myocytes from ischemic damage [42]. All these results lend support to the hypothesis that exercise training-induced cardioprotection to I/R is not only accompanied by the overexpression of caveolin 3, but also strictly related to it by a cause-effect relationship.

In order to identify functional gene sets correlated with mild chronic exercise training, we applied GSA analysis. GSA analysis identified only 2 gene sets correlated with exercise training: the ARENRF2PATHWAY and the GABAPATHWAY. There are some evidences that physical exercise leads to the enhanced transient formation of reactive oxygen species (ROS) [14]. Oxygen radicals are important intracellular second messengers that mediate cardioprotection [43]. Increased levels of ROS generated in working muscles favor a change in the redox-balance towards a more pro-oxidant state counteracted by a complex network of antioxidant systems [44], as suggested by the association of exercise with the "Oxidative stress induced gene expression via Nrf2" pathway. The functional consequences of exercise-induced oxidative stress are only partly understood; nevertheless, the present data demonstrated that our protocol is able to induce cardioprotection, as evaluated by functional I/R experiments. In addition, the association of the GABA pathway with training is consistent with the role of GABA receptor activity in the cardioprotection induced by ischemic preconditioning [43]. The association of these two pathways is consistent with our data on Cav3 upregulation: in fact, several data showed that caveolin 3 protein plays an important role in ROS response through MAP kinases mediated intracellular signal transduction underlying cardioprotection [45,46].

Even if we confirmed by other molecular biology approaches the coherence of our microarray data, this study have the limitations of all transcriptomics approaches concerning the post-transcriptional and post-transductional behavior of the differentially expressed genes and the possibility of type I (false positive genes) and type II (false negative genes) statistical error due to the high number of parameters investigated in each experiments.

## Conclusion

In conclusion, mild aerobic chronic exercise training, at a level that does not induce cardiac hypertrophy, was found to induce cardioprotection toward I/R events. Even if our results underline the finding that gene expression profiles of left ventricles from sedentary and trained rats were very similar, in spite of the persistence of exercise-induced cardioprotection, the fact that few genes appear to be overexpressed in LV of trained animals suggests that they may be involved in relevant mechanisms of cardioprotection. While further global transcriptomic and proteomic profiling, carried out during and at different times after bout of exercise, may help to better understand the molecular mechanisms underlying the beneficial effect of mild exercise training, the present results clearly show a link between the long lasting expression of some exercise training-induced genes and cardioprotection.

## Competing interests

The authors declare that they have no competing interests.

## Authors' contributions

BG, RA, MM, MS, AV conceived the study, and participated in its design and coordination. LR, IL, RL, SDT, VM, FE carried out the training of animals, the gene expression profiling, the real time PCR, the western blot and immunohistochemistry analysis, and the ischemia-reperfusion studies. AM, LR, IL, AC performed the microarray data extraction, statistical analysis and interpretation of data. BG, MB, MM, MS drafted the manuscript or revised it critically for important intellectual content. All authors read and approved the final manuscript.

## Supplementary Material

Additional file 1 **Real Time PCR: primer sequences, size of the amplification products and mean efficiency of the amplification reactions**. In this table primer sequences, size of the amplification products and mean efficiency of the amplification reactions of real time PCR are reported. Click here for file

Additional file 2 **Full list of the 300 most expressed genes independent of the rat group (TRA AND CTR)**. In this table, the full list of the 300 most expressed genes in the overall animal population (n = 10 CTR + n = 10 TRA) is reported. Click here for file

Additional file 3 **Gene ontology (GO) analysis for biological processes**. In this table, the gene ontology analysis according to biological processes of the 300 most expressed genes in the overall animal population is reported. The genes associated with each biological process among the 300 most expressed genes are reported. Click here for file

Additional file 4 **Gene ontology (GO) analysis for molecular functions**. In this table, the gene ontology analysis according to molecular functions of the 300 most expressed genes in the overall animal population is reported. The genes associated with each molecular function among the 300 most expressed genes are reported. Click here for file
