# Supplementary material for: Gene expression profile of rat left ventricles reveals persisting changes following chronic mild exercise protocol: implications for cardioprotection
Source: BMC Genomics. 2009 Jul 30;10:342. doi: 10.1186/1471-2164-10-342 (PMC2907697; doi:10.1186/1471-2164-10-342)
Supplement: Additional file 1 — Real Time PCR: primer sequences, size of the amplification products and mean efficiency of the amplification reactions. In this table primer sequences, size of the amplification products and mean efficiency of the amplification reactions of real time PCR are reported. [file 1471-2164-10-342-S1.doc]

**Additional file 1.** Real Time PCR: primer sequences, size of the amplification products and mean efficiency of the amplification reactions.

| **Gene name** | **Unigene no.** | **Left primer** | **Right primer** | **Amplicon length**  **(bp)** | **PCR efficiency** |
| --- | --- | --- | --- | --- | --- |
| **RPL-13alpha** | Rn 92211 | GATGAACACCAACCCGTCTC | CACCATCCGCTTTTTCTTGT | 175 | 1.946 |
| **Cav-3** | Rn 98191 | GCTGCACCAGGGAACACTAT | CCCTATTTATGCCCAGCAAA | 161 | 1.922 |
| **Eno-3** | Rn 197559 | GCTGATCTTGTGGTGGGACT | CTTCTCATTTGGCCTTTGGA | 168 | 1.845 |
| **Hif-1alpha** | Rn 10852 | AAGTCAGCAACGTGGAAGGT | CGTCATAGGCGGTTTCTTGT | 104 | 1.795 |
| **Similar to C11orf17 protein (RGD1306959)** | Rn 106344 | TTGTCTACACCGGAGGAAGG | ATGCTTCCTGGACCAACAGA | 188 | 1.890 |
