# Supplementary material for: Gene expression profile of rat left ventricles reveals persisting changes following chronic mild exercise protocol: implications for cardioprotection
Source: BMC Genomics. 2009 Jul 30;10:342. doi: 10.1186/1471-2164-10-342 (PMC2907697; doi:10.1186/1471-2164-10-342)
Supplement: Additional file 2 — Full list of the 300 most expressed genes independent of the rat group (TRA AND CTR). In this table, the full list of the 300 most expressed genes in the overall animal population (n = 10 CTR + n = 10 TRA) is reported. [file 1471-2164-10-342-S2.doc]

**FULL LIST OF THE 300 MOST EXPRESSED GENES INDEPENDENT OF THE RAT GROUP (TRA AND CTR)**

Probe name=Affymetrix number of the probe set that recognized the specific transcript.

| **Probe name** | **GenBank** | **Gene ID** | **Gene Symbol** | **Gene Names** |
| --- | --- | --- | --- | --- |
| 1375107_at | AI170758 | NA | NA | NA |
| 1389956_a_at | AI180257 | NA | NA | NA |
| 1375651_at | AI230164 | NA | NA | NA |
| 1375340_at | AI104151 | NA | NA | NA |
| 1388200_at | BF419995 | 363925 | LOC363925 | NA |
| 1370856_at | AA800705 | 29275 | Actc1 | actin alpha cardiac 1 |
| 1388159_at | AA849795 | NA | NA | NA |
| 1368093_at | NM_017239 | 29556 | Myh6 | myosin heavy chain, polypeptide 6 |
| 1398248_s_at | NM_017240 | 29557 | Myh7 | myosin, heavy polypeptide 7, cardiac muscle, beta |
| 1370239_at | AI179404 | 25632 | Hba-a1 | hemoglobin alpha, adult chain 1 |
| 1386931_at | NM_017144 | 29248 | Tnni3 | troponin I, cardiac |
| 1367592_at | NM_012676 | 24837 | Tnnt2 | troponin T2, cardiac |
| 1369065_a_at | NM_017290 | 29693 | Atp2a2 | ATPase, Ca++ transporting, cardiac muscle, slow twitch 2 |
| 1367660_at | NM_024162 | 79131 | Fabp3 | fatty acid binding protein 3 |
| 1370240_x_at | AI179404 | 25632 | Hba-a1 | hemoglobin alpha, adult chain 1 |
| 1388112_at | BG666999 | 85333 | Slc25a4 | solute carrier family 25 (mitochondrial adenine nucleotide translocator) member 4 |
| 1367572_at | NM_012606 | 24585 | Myl3 | myosin, light polypeptide 3 |
| 1368724_a_at | NM_019131 | 24851 | Tpm1 | tropomyosin 1, alpha |
| 1387768_at | NM_021588 | 59108 | Mb | myoglobin |
| 1367782_at | NM_012812 | 25278 | Cox6a2 | cytochrome c oxidase, subunit VIa, polypeptide 2 |
| 1370026_at | NM_012935 | 25420 | Cryab | crystallin, alpha B |
| 1375108_at | AI008105 | NA | NA | NA |
| 1387890_at | AI229633 | 25348 | Rps29 | ribosomal protein S29 |
| 1367557_s_at | NM_017008 | 24383 | Gapd | glyceraldehyde-3-phosphate dehydrogenase |
| 1371241_x_at | AF370889 | 24851 | Tpm1 | tropomyosin 1, alpha |
| 1370218_at | AA848319 | 24534 | Ldhb | lactate dehydrogenase B |
| 1371354_at | AI710682 | 290561 | Tncc_predicted | troponin C, cardiac/slow skeletal (predicted) |
| 1370277_at | M23984 | 245959 | Slc25a3 | solute carrier family 25 (mitochondrial carrier |
| 1367739_at | NM_012786 | 25250 | Cox8h | Cytochrom c oxidase subunit VIII-H (heart/muscle) |
| AFFX-r2-P1-cre-3_at | AFFX-R2-P1-CRE-3 | NA | NA | NA |
| 1370288_a_at | AF372216 | 24851 | Tpm1 | tropomyosin 1, alpha |
| 1367617_at | NM_012495 | 24189 | Aldoa | aldolase A |
| AFFX_Rat_GAPDH_3_at | AFFX_RAT_GAPDH_3 | 24383 | Gapd | glyceraldehyde-3-phosphate dehydrogenase |
| 1388403_at | AI172491 | 361596 | LOC361596 | NA |
| 1367607_at | NM_017202 | 29445 | Cox4i1 | cytochrome c oxidase subunit IV isoform 1 |
| 1370275_at | M19044 | 171374 | Atp5b | ATP synthase, H+ transporting, mitochondrial F1 complex, beta polypeptide |
| 1367565_a_at | NM_012848 | 25319 | Fth1 | ferritin, heavy polypeptide 1 |
| 1370157_at | BI290034 | 64672 | Pln | phospholamban |
| 1388349_at | AA799557 | 117001 | Ckmt2 | creatine kinase, mitochondrial 2, sarcomeric |
| 1388608_x_at | AI577319 | 25632 | Hba-a1 | hemoglobin alpha, adult chain 1 |
| 1370918_a_at | BI275939 | 116550 | Atp5c1 | ATP synthase, H+ transporting, mitochondrial F1 complex, gamma polypeptide 1 |
| 1367620_at | NM_053756 | 114630 | Atp5g3 | ATP synthase, H+ transporting, mitochondrial F0 complex, subunit c (subunit 9) isoform 3 |
| 1371701_at | BG666921 | 299954 | Ndufb9_predicted | NADH dehydrogenase (ubiquinone) 1 beta subcomplex, 9 (predicted) |
| 1367626_at | NM_012530 | 24265 | Ckm | creatine kinase, muscle |
| 1387898_at | D29960 | 192245 | Hspb6 | heat shock protein, alpha-crystallin-related, B6 |
| 1367629_at | NM_022503 | 29507 | Cox7a3 | cytochrome c oxidase, subunit 7a 3 |
| 1388876_at | AI231802 | 64672 | Pln | phospholamban |
| AFFX_Rat_GAPDH_M_at | AFFX_RAT_GAPDH_M | 24383 | Gapd | glyceraldehyde-3-phosphate dehydrogenase |
| 1371335_at | AA944393 | 300677 | RGD:1303259 | similar to CG6105-PA |
| 1369939_at | AI104240 | 25309 | Cycs | cytochrome c, somatic |
| AFFX-r2-P1-cre-5_at | AFFX-R2-P1-CRE-5 | NA | NA | NA |
| 1374426_at | BG670074 | 362897 | Uqcrb_predicted | ubiquinol-cytochrome c reductase binding protein (predicted) |
| 1370284_at | AF010323 | 245958 | Atp5e | ATP synthase, H+ transporting, mitochondrial F1 complex, epsilon subunit |
| 1367665_at | L81174 | 27064 | Ankrd1 | ankyrin repeat domain 1 (cardiac muscle) |
| 1367576_at | S41066 | 24404 | Gpx1 | glutathione peroxidase 1 |
| 1370378_at | J05266 | 65262 | Atp5a1 | ATP synthase, H+ transporting, mitochondrial F1 complex, alpha subunit, isoform 1. |
| 1370276_at | D13127 | 192241 | Atp5o | ATP synthase, H+ transporting, mitochondrial F1 complex, O subunit |
| 1386993_at | NM_017240 | 29557 | Myh7 | myosin, heavy polypeptide 7, cardiac muscle, beta |
| 1371801_at | AI104354 | 295426 | Myoz2_predicted | myozenin 2 (predicted) |
| 1367664_at | NM_013220 | 27064 | Ankrd1 | ankyrin repeat domain 1 (cardiac muscle) |
| 1398326_at | BI282332 | 361824 | RGD:1359417 | similar to Nur77 downstream protein 2 |
| 1367583_at | NM_053867 | 116646 | Tpt1 | tumor protein, translationally-controlled 1 |
| 1393245_at | AI029057 | 114209 | Phyh | phytanoyl-CoA hydroxylase |
| 1374352_at | AI711147 | 29275 | Actc1 | actin alpha cardiac 1 |
| 1367653_a_at | NM_033235 | 24551 | Mdh1 | malate dehydrogenase 1, NAD (soluble) |
| 1386852_x_at | D16554 | 192255 | Ubb | polyubiquitin |
| 1367622_at | NM_019383 | 56080 | Atp5h | ATP synthase, H+ transporting, mitochondrial F0 complex, subunit d |
| 1370213_at | BI282111 | 29206 | Nsep1 | nuclease sensitive element binding protein 1 |
| 1372649_at | BM390305 | 50565 | Hspb7 | heat shock 27kD protein family, member 7 (cardiovascular) |
| 1367757_at | NM_019360 | 54322 | Cox6c | cytochrome c oxidase, subunit VIc |
| 1398855_at | AA893531 | 171375 | Atp5f1 | ATP synthase, H+ transporting, mitochondrial F0 complex, subunit b, isoform 1 |
| 1374353_x_at | AI711147 | 29275 | Actc1 | actin alpha cardiac 1 |
| 1388301_at | BI287993 | 301011 | RGD:1303314 | ubiquinol-cytochrome c reductase core protein I |
| 1387271_at | NM_053674 | 114209 | Phyh | phytanoyl-CoA hydroxylase |
| 1386965_at | NM_012598 | 24539 | Lpl | lipoprotein lipase |
| 1367735_at | NM_012819 | 25287 | Acadl | acetyl-Coenzyme A dehydrogenase, long-chain |
| 1371417_at | AI233054 | 497902 | Qpc | NA |
| 1370238_at | AA891707 | NA | NA | NA |
| 1370888_at | X15030 | 252934 | Cox5a | cytochrome c oxidase, subunit Va |
| 1386887_at | NM_053586 | 94194 | Cox5b | cytochrome c oxidase subunit Vb |
| 1371381_at | AI007981 | 306117 | LOC306117 | NA |
| 1367599_at | NM_017311 | 29754 | Atp5g1 | ATP synthase, H+ transporting, mitochondrial F0 complex, subunit c (subunit 9), isoform 1 |
| 1371504_at | BF281215 | 500547 | LOC500547 | NA |
| 1388304_at | BI281307 | 294964 | Ndufb5_predicted | NADH dehydrogenase (ubiquinone) 1 beta subcomplex, 5 (predicted) |
| 1371297_at | BI281697 | 170704 | Hrh4 | histamine H4 receptor |
| 1371371_at | AW434041 | 503438 | LOC503438 | NA |
| 1371398_at | BF281400 | NA | NA | NA |
| 1367553_x_at | NM_033234 | 24440 | Hbb | hemoglobin beta chain complex |
| 1371323_at | BG666002 | NA | NA | NA |
| 1367604_at | NM_022501 | 338401 | RGD:1302959 | cysteine-rich protein 2 |
| 1371387_at | AA866477 | 303393 | Cox7b | cytochrome c oxidase subunit VIIb |
| 1372123_at | AI172320 | 298596 | Sdhb_predicted | succinate dehydrogenase complex, subunit B, iron sulfur (Ip) (predicted) |
| 1367589_at | NM_024398 | 79250 | Aco2 | aconitase 2, mitochondrial |
| 1371321_at | AI230604 | 502318 | LOC502318 | NA |
| 1372595_at | BF284889 | 291245 | Actn2_predicted | actinin alpha 2 (predicted) |
| 1370006_at | NM_019223 | 29478 | Ndufs6 | NADH dehydrogenase (ubiquinone) Fe-S protein 6 |
| 1387121_a_at | NM_133583 | 171114 | Ndrg2 | N-myc downstream regulated gene 2 |
| 1398243_at | NM_057144 | 117505 | Csrp3 | cysteine-rich protein 3 |
| 1367645_at | NM_017152 | 29286 | Rps17 | ribosomal protein S17 |
| AFFX_Rat_GAPDH_5_at | AFFX_RAT_GAPDH_5 | 24383 | Gapd | glyceraldehyde-3-phosphate dehydrogenase |
| 1367606_at | NM_017153 | 29288 | Rps3a | ribosomal protein S3a |
| 1388303_at | AI598536 | 498998 | LOC498998 | NA |
| 1375219_a_at | AA944861 | 292812 | LOC292812 | NA |
| 1371605_at | AI710281 | 299739 | RGD1311462_predicted | similar to RIKEN cDNA 2410011G03 (predicted) |
| 1385797_at | BF525047 | 29275 | Actc1 | actin alpha cardiac 1 |
| 1369897_s_at | BI277035 | 24896 | Gnas | GNAS complex locus |
| 1367630_at | NM_031110 | 81774 | Rps11 | ribosomal protein S11 |
| 1386902_at | NM_031355 | 83532 | Vdac3 | voltage-dependent anion channel 3 |
| 1398240_at | NM_024351 | 24468 | Hspa8 | heat shock protein 8 |
| 1371342_at | BI277021 | 300047 | Cyc1_predicted | cytochrome c-1 (predicted) |
| 1388391_at | AA996544 | 363441 | LOC363441 | NA |
| 1367702_at | NM_016986 | 24158 | Acadm | acetyl-Coenzyme A dehydrogenase, medium chain |
| 1367610_at | NM_031103 | 81767 | Rpl19 | ribosomal protein L19 |
| 1389964_at | AA944343 | 293453 | Ndufab1_predicted | NADH dehydrogenase (ubiquinone) 1, alpha/beta subcomplex, 1 (predicted) |
| 1369966_a_at | BG375811 | 81776 | Rps24 | ribosomal protein S24 |
| 1367595_s_at | NM_012512 | 24223 | B2m | beta-2 microglobulin |
| 1375518_at | AI104533 | 84015 | Ttn | titin |
| 1390020_at | BI277513 | 360975 | LOC360975 | NA |
| 1369313_at | NM_031677 | 63839 | Fhl2 | four and a half LIM domains 2 |
| AFFX-CreX-3_at | AFFX-CREX-3 | NA | NA | NA |
| 1398324_at | BI279866 | 499057 | LOC499057 | NA |
| 1368211_at | NM_022672 | 29284 | Rps14 | ribosomal protein S14 |
| 1371041_at | M22756 | 81728 | Ndufv2 | NADH dehydrogenase (ubiquinone) flavoprotein 2 |
| 1369927_at | NM_031151 | 81829 | Mor1 | malate dehydrogenase, mitochondrial |
| 1386901_at | NM_031561 | 29184 | Cd36 | cd36 antigen |
| 1367694_at | NM_133618 | 171155 | Hadhb | hydroxyacyl-Coenzyme A dehydrogenase/3-ketoacyl-Coenzyme A thiolase/enoyl-Coenzyme A hydratase (trifunctional protein), beta subunit |
| 1371951_at | AA800031 | 63839 | Fhl2 | four and a half LIM domains 2 |
| 1367784_a_at | AF314657 | 24854 | Clu | clusterin |
| 1386880_at | NM_130433 | 170465 | Acaa2 | acetyl-Coenzyme A acyltransferase 2 (mitochondrial 3-oxoacyl-Coenzyme A thiolase) |
| 1367717_at | NM_053597 | 94266 | Rps27 | ribosomal protein S27 |
| 1387773_at | NM_012839 | 25309 | Cycs | cytochrome c, somatic |
| 1371254_at | AI179382 | 291103 | Uqcrfs1 | ubiquinol-cytochrome c reductase, Rieske iron-sulfur polypeptide 1 |
| 1371245_a_at | BI287300 | NA | NA | NA |
| 1371415_at | BI279016 | 366448 | Uqcrh_predicted | ubiquinol-cytochrome c reductase hinge protein (predicted) |
| 1387883_a_at | BG668902 | 81814 | Tmsb4x | thymosin, beta 4 |
| 1373130_at | AI407239 | 306616 | Myom2 | myomesin 2 |
| 1371302_at | AI406651 | 311358 | LOC311358 | NA |
| 1367586_at | NM_017025 | 24533 | Ldha | lactate dehydrogenase A |
| 1382088_at | AI548753 | NA | NA | NA |
| 1386867_at | NM_133561 | 171087 | Brp44l | brain protein 44-like |
| 1371301_at | BG666892 | 29257 | Rpl9 | ribosomal protein L9 |
| 1371483_at | AA891872 | 310378 | Nnt | nicotinamide nucleotide transhydrogenase |
| 1398767_at | NM_017314 | 50522 | Ubc | ubiquitin C |
| 1367625_at | NM_031100 | 81764 | Rpl10 | ribosomal protein L10 |
| 1388597_at | AI104571 | 295929 | Mybpc3_predicted | myosin binding protein C, cardiac (predicted) |
| 1367706_at | NM_031353 | 83529 | Vdac1 | voltage-dependent anion channel 1 |
| 1388506_at | AW144509 | 306871 | Dsp_predicted | desmoplakin (predicted) |
| 1367951_at | NM_017328 | 24959 | Pgam2 | phosphoglycerate mutase 2 |
| 1370274_at | D16554 | 192255 | Ubb | polyubiquitin |
| 1398789_at | NM_031106 | 81770 | Rpl37 | ribosomal protein L37 |
| 1389820_at | BF289041 | NA | NA | NA |
| 1388315_at | BI282661 | 299310 | LOC299310 | NA |
| 1371554_at | AA799471 | NA | NA | NA |
| AFFX-CreX-5_at | AFFX-CREX-5 | NA | NA | NA |
| 1388310_at | AI012372 | 500402 | LOC500402 | NA |
| 1371307_at | AW914090 | 140661 | Rplp1 | ribosomal protein, large, P1 |
| 1372002_at | AI411352 | 24392 | Gja1 | gap junction membrane channel protein alpha 1 |
| 1386885_at | NM_022594 | 64526 | Ech1 | enoyl coenzyme A hydratase 1, peroxisomal |
| 1367687_a_at | M25719 | 25508 | Pam | peptidylglycine alpha-amidating monooxygenase |
| 1371308_at | AA799501 | 29426 | Rps4x | ribosomal protein S4, X-linked |
| 1371295_at | AW914097 | 122772 | Rps20 | ribosomal protein S20 |
| 1373362_at | BI296109 | 293448 | RGD:1359150 | ubiquinol-cytochrome c reductase core protein II |
| 1367634_at | NM_022506 | 64298 | Rpl31 | ribosomal protein L31 |
| 1369933_at | AF268468 | 83531 | Vdac2 | voltage-dependent anion channel 2 |
| 1371421_at | BM391873 | 502508 | LOC502508 | NA |
| 1370230_at | BG666602 | 94271 | Atp5j | ATP synthase, H+ transporting, mitochondrial F0 complex, subunit F6 |
| 1384548_at | BM388159 | 28298 | Rpl32 | ribosomal protein L32 |
| 1370165_at | AI406565 | 84416 | Smpx | small muscle protein, X-linked |
| 1375295_at | AI009657 | 170587 | Cs | citrate synthase |
| 1371340_at | AW919054 | 140662 | Rplp2 | ribosomal protein, large P2 |
| 1367600_at | NM_022531 | 64362 | Des | desmin |
| 1369928_at | NM_019212 | 29437 | Acta1 | actin, alpha 1, skeletal muscle |
| 1388323_at | BG381033 | 362440 | Ndufa9_predicted | NADH dehydrogenase (ubiquinone) 1 alpha subcomplex, 9 (predicted) |
| 1371482_at | AA851675 | 289218 | Ndufs2_predicted | NADH dehydrogenase (ubiquinone) Fe-S protein 2 (predicted) |
| 1373041_at | AI555535 | 301427 | Ndufb3_predicted | NADH dehydrogenase (ubiquinone) 1 beta subcomplex 3 (predicted) |
| 1389866_at | AI599296 | 499187 | LOC499187 | NA |
| 1383161_a_at | AI008646 | NA | NA | NA |
| 1377720_x_at | AA892765 | NA | NA | NA |
| 1367573_at | NM_017160 | 29304 | Rps6 | ribosomal protein S6 |
| 1370865_at | BI277627 | 25179 | Idh3g | isocitrate dehydrogenase 3, gamma |
| 1371912_at | AI231358 | 362837 | Ndufs7_predicted | NADH dehydrogenase (ubiquinone) Fe-S protein 7 (predicted) |
| 1375197_at | BG665384 | NA | NA | NA |
| 1398830_at | NM_022697 | 64638 | Rpl28 | ribosomal protein L28 |
| 1367561_at | NM_022514 | 64306 | Rpl27 | ribosomal protein L27 |
| 1371380_at | AI411413 | 29554 | Pdha1 | pyruvate dehydrogenase E1 alpha 1 |
| 1367640_at | NM_031709 | 65139 | Rps12 | ribosomal protein S12 |
| 1377719_a_at | AA892765 | NA | NA | NA |
| 1387049_at | BI296041 | 29556 | Myh6 | myosin heavy chain, polypeptide 6 |
| 1371327_a_at | BG666668 | 295810 | LOC295810 | NA |
| 1388114_at | X05566 | 50685 | Mrlcb | myosin light chain, regulatory B |
| 1388375_at | AI234096 | 25725 | Prkar1a | protein kinase, cAMP dependent regulatory, type I, alpha |
| 1398871_at | BG671311 | 291434 | RGD:1303019 | ribosomal protein L17 |
| 1370956_at | BM390253 | 29139 | Dcn | decorin |
| 1371416_at | AI231746 | 293655 | RGD:1359247 | NADH dehydrogenase (ubiquinone) flavoprotein 1, 51kDa |
| 1371414_at | BI285576 | 296654 | RGD:1303089 | gelsolin |
| 1367568_a_at | NM_012862 | 25333 | Mgp | matrix Gla protein |
| 1370278_at | U00926 | 245965 | Atp5d | ATP synthase, H+ transporting, mitochondrial F1 complex, delta subunit |
| 1371311_at | AI009817 | 289217 | RGD:1359454 | succinate dehydrogenase complex, subunit C |
| 1370242_at | AA848284 | 124323 | Rps23 | ribosomal protein S23 |
| 1398761_at | NM_031099 | 81763 | Rpl5 | ribosomal protein L5 |
| 1398774_at | NM_022699 | 64640 | Rpl30 | ribosomal protein L30 |
| 1388296_at | BF281388 | 294282 | Rps18 | ribosomal protein S18 |
| 1371344_at | AI177054 | 499133 | LOC499133 | NA |
| 1398917_at | BF281221 | 297755 | Rpl7 | ribosomal protein L7 |
| 1388343_at | AI575943 | 361385 | Ndufb7_predicted | NADH dehydrogenase (ubiquinone) 1 beta subcomplex, 7 (predicted) |
| 1372241_at | AI411388 | 25502 | Oaz1 | ornithine decarboxylase antizyme 1 |
| 1367678_at | NM_130428 | 157074 | Sdha | succinate dehydrogenase complex, subunit A, flavoprotein (Fp) |
| 1374816_at | AI103939 | 363091 | RGD:1359197 | similar to hypothetical protein FLJ30973 |
| 1367819_at | NM_013177 | 25721 | Got2 | glutamate oxaloacetate transaminase 2 |
| 1388160_a_at | AI171793 | 94173 | Idh3B | isocitrate dehydrogenase 3 (NAD+) beta |
| 1375336_at | AI237389 | 311039 | Galnt13 | UDP-N-acetyl-alpha-D-galactosamine:polypeptide N-acetylgalactosaminyltransferase 13 |
| 1367597_at | NM_031706 | 65136 | Rps8 | ribosomal protein S8 |
| 1388414_at | AI410498 | 595134 | LOC595134 | NA |
| 1368988_at | AW520914 | 29209 | Casq2 | calsequestrin 2 |
| 1392530_at | AI409545 | NA | NA | NA |
| 1388110_at | X61043 | 171361 | Eef1a1 | eukaryotic translation elongation factor 1 alpha 1 |
| 1371316_at | AI177362 | 29752 | Fau | Finkel-Biskis-Reilly murine sarcoma virusubiquitously expressed |
| 1388318_at | BI279760 | 24644 | Pgk1 | phosphoglycerate kinase 1 |
| 1388570_at | AI232494 | 361365 | Polr2c_predicted | polymerase (RNA) II (DNA directed) polypeptide C (predicted) |
| 1377499_a_at | BF420810 | 292905 | Hrc | histidine rich calcium binding protein |
| 1370678_s_at | D00688 | 29253 | Maoa | monoamine oxidase A |
| 1368566_a_at | AA964381 | 64539 | RGD:621020 | MIPP65 protein |
| 1398754_at | NM_031687 | 64156 | Uba52 | ubiquitin A-52 residue ribosomal protein fusion product 1 |
| 1375349_at | BI295776 | NA | NA | NA |
| 1371319_at | BI278308 | 290364 | Itm2b | integral membrane protein 2B |
| 1371933_at | AI228039 | 64183 | Pde4dip | phosphodiesterase 4D interacting protein (myomegalin) |
| 1367603_at | NM_022922 | 24849 | Tpi1 | triosephosphate isomerase 1 |
| 1387957_a_at | AF255888 | 84357 | Sh3kbp1 | SH3-domain kinase binding protein 1 |
| 1367641_at | NM_017050 | 24786 | Sod1 | superoxide dismutase 1 |
| 1369931_at | NM_053297 | 25630 | Pkm2 | pyruvate kinase, muscle |
| 1388489_at | AA875107 | NA | NA | NA |
| 1388358_at | AW252650 | 292845 | RGD:1303312 | electron-transfer-flavoprotein, beta polypeptide |
| 1367560_at | NM_022402 | 64205 | Arbp | acidic ribosomal phosphoprotein P0 |
| 1371577_at | AI171362 | 301458 | RGD:1359670 | NADH dehydrogenase (ubiquinone) Fe-S protein 1, 75kDa |
| 1372475_at | AI410877 | 298575 | Pink1_predicted | PTEN induced putative kinase 1 (predicted) |
| 1386871_at | NM_017165 | 29328 | Gpx4 | glutathione peroxidase 4 |
| 1371102_x_at | X05080 | 24440 | Hbb | hemoglobin beta chain complex |
| 1388364_at | BG381650 | 295923 | Ndufs3_predicted | NADH dehydrogenase (ubiquinone) Fe-S protein 3 (predicted) |
| 1387019_at | NM_080481 | 140608 | Atp5i | ATP synthase, H+ transporting, mitochondrial F0 complex, subunit e |
| 1371312_at | AA799736 | 316643 | Chchd2_predicted | coiled-coil-helix-coiled-coil-helix domain containing 2 (predicted) |
| 1398775_at | NM_053982 | 117053 | Rps15a | ribosomal protein S15a |
| 1370982_at | BI275633 | 24701 | Pygm | muscle glycogen phosphorylase |
| 1371377_at | BG668512 | 305202 | LOC305202 | NA |
| 1388327_at | AI232357 | 294362 | RGD1309529_predicted | similar to DNA segment, Chr 10, ERATO Doi 214, expressed (predicted) |
| 1398872_at | BE107613 | 161477 | Rps13 | ribosomal protein S13 |
| 1371318_at | BG666872 | 140655 | Rps16 | ribosomal protein S16 |
| 1371305_at | AI409193 | 296580 | LOC296580 | NA |
| 1371320_at | BF282337 | 290364 | Itm2b | integral membrane protein 2B |
| 1388362_at | BM390197 | 314759 | LOC314759 | NA |
| 1367685_at | NM_031113 | 81777 | Rps27a | ribosomal protein S27a |
| AFFX-r2-Ec-bioD-3_at | AFFX-R2-EC-BIOD-3 | NA | NA | NA |
| 1371313_at | AI172199 | 499523 | LOC499523 | NA |
| 1367764_at | NM_012923 | 25405 | Ccng1 | cyclin G1 |
| 1398885_at | AA925327 | 29282 | Rpl23 | ribosomal protein L23 |
| 1370237_at | AA799574 | 113965 | Hadhsc | L-3-hydroxyacyl-Coenzyme A dehydrogenase, short chain |
| 1373920_at | AI175534 | 316085 | RGD1307844_predicted | similar to 106 kDa O-GlcNAc transferase-interacting protein (predicted) |
| 1367639_a_at | U92700 | 83789 | Rps2 | ribosomal protein S2 |
| 1375632_at | H35210 | 502741 | LOC502741 | NA |
| 1386858_at | NM_031101 | 81765 | Rpl13 | ribosomal protein L13 |
| 1388372_at | AW914118 | 296709 | Rpl35 | ribosomal protein L35 |
| 1367799_at | NM_012660 | 24799 | Stnl | statin-like |
| 1371761_at | AI013910 | 362041 | Rpl34_predicted | ribosomal protein L34 (predicted) |
| 1388294_at | AI176608 | 363061 | Sdhd | succinate dehydrogenase complex, subunit D, integral membrane protein |
| AFFX_Rat_beta-actin_3_at | AFFX_RAT_BETA-ACTIN_3 | 81822 | Actb | actin, beta |
| 1375516_at | AA891171 | 293130 | Ndufc2_predicted | NADH dehydrogenase (ubiquinone) 1, subcomplex unknown, 2 (predicted) |
| 1370296_at | M34728 | 25541 | Scp2 | sterol carrier protein 2 |
| 1398760_at | NM_021264 | 57809 | Rpl35a | ribosomal protein L35a |
| 1386898_at | NM_012966 | 25462 | Hspe1 | heat shock 10 kDa protein 1 |
| 1376789_at | AI410679 | 291926 | RGD1305801_predicted | similar to Myosin light chain kinase 2, skeletal/cardiac muscle (MLCK2) (predicted) |
| 1367663_at | NM_017264 | 29630 | Psme1 | protease (prosome, macropain) 28 subunit, alpha |
| 1371330_at | BI284252 | 362631 | Rpl11_predicted | ribosomal protein L11 (predicted) |
| 1370939_at | D90109 | 25288 | Acsl1 | acyl-CoA synthetase long-chain family member 1 |
| 1373915_at | AI044427 | 308405 | Dm15_predicted | dystrophia myotonica kinase, B15 (predicted) |
| 1371355_at | BI280270 | 296658 | Ndufa8_predicted | NADH dehydrogenase (ubiquinone) 1 alpha subcomplex, 8 (predicted) |
| 1375411_at | BI277002 | 299643 | Ndufa7_predicted | NADH dehydrogenase (ubiquinone) 1 alpha subcomplex, 7 (B14.5a) (predicted) |
| 1389066_at | BI274408 | 140666 | Dscr1l1 | Down syndrome critical region gene 1-like 1 |
| 1367578_at | NM_017169 | 29338 | Prdx2 | peroxiredoxin 2 |
| 1370180_at | AA891213 | 94267 | Nudt4 | nudix (nucleoside diphosphate linked moiety X)-type motif 4 |
| 1386894_at | NM_022229 | 63868 | Hspd1 | heat shock protein 1 (chaperonin) |
| 1398827_at | NM_013087 | 25621 | Cd81 | CD 81 antigen |
| 1370866_at | AA944073 | 124440 | Rpl41 | ribosomal protein L41 |
| 1373987_at | AI410448 | 56817 | Kcnip2 | Kv channel-interacting protein 2 |
| 1367766_at | NM_031833 | 83782 | Nme2 | expressed in non-metastatic cells 2 |
| 1371954_at | BF290193 | 301509 | LOC301509 | NA |
| 1367623_at | NM_031102 | 81766 | Rpl18 | ribosomal protein L18 |
| 1367588_a_at | NM_022179 | 25060 | Hk3 | hexokinase 3 |
| 1372737_at | BF395095 | 498587 | LOC498587 | NA |
| 1390019_at | BM390456 | 117056 | RGD:621095 | H3 histone, family 3B |
| 1367679_at | NM_013069 | 25599 | Cd74 | CD74 antigen (invariant polpypeptide of major histocompatibility class II antigen-associated) |
| 1367596_at | NM_013224 | 27139 | Rps26 | ribosomal protein S26 |
| 1398882_at | BI282255 | 25538 | Rps5 | ribosomal protein S5 |
| 1383162_at | AI008646 | NA | NA | NA |
| 1375119_at | BI284798 | 25489 | Nedd4a | neural precursor cell expressed, developmentally down-regulated gene 4A |
| 1386891_at | NM_017236 | 29542 | Pbp | phosphatidylethanolamine binding protein |
| 1372296_at | AA800892 | 498066 | LOC498066 | NA |
| 1379243_at | AA819547 | 315167 | Ndufa6_predicted | NADH dehydrogenase (ubiquinone) 1 alpha subcomplex, 6 (B14) (predicted) |
| 1373193_at | BE108670 | NA | NA | NA |
| 1370109_s_at | NM_033539 | 29652 | Eef1a2 | eukaryotic translation elongation factor 1 alpha 2 |
| 1367709_at | NM_017125 | 29186 | Cd63 | CD63 antigen |
| 1371642_at | AI169170 | 303831 | Eif4a2_predicted | eukaryotic translation initiation factor 4A2 (predicted) |
