# Supplementary material for: Gene expression profile of rat left ventricles reveals persisting changes following chronic mild exercise protocol: implications for cardioprotection
Source: BMC Genomics. 2009 Jul 30;10:342. doi: 10.1186/1471-2164-10-342 (PMC2907697; doi:10.1186/1471-2164-10-342)
Supplement: Additional file 3 — Gene ontology (GO) analysis for biological processes. In this table, the gene ontology analysis according to biological processes of the 300 most expressed genes in the overall animal population is reported. The genes associated with each biological process among the 300 most expressed genes are reported. [file 1471-2164-10-342-S3.doc]

**GENE ONTOLOGY (GO) ANALYSIS FOR BIOLOGICAL PROCESSES.**

**n=number of genes associated with the biological process among the 300 most expressed genes.**

**N=number of genes associated with the biological process present in the GeneChip Rat Genome 230 v2.0 Array.**

**P value = Fisher exact test p value**

| **GO ID** | **n** | **N** | **P value** | **GO Names** | **Gene Symbol** |
| --- | --- | --- | --- | --- | --- |
| GO:0006119 | 22 | 32 | 6.25E-28 | oxidative phosphorylation | Cox8h, Atp5c1, Ndufb9_predicted, MGC72942, Uqcrb_predicted, Atp5e, Uqcrc1, Ndufb5_predicted, Ndufb4, Ndufs6, Cyc1_predicted, Ndufv2, Uqcrh, Ndufs2, Ndufb3_predicted, Ndufs7, Ndufv1, Atp5d, Ndufs5b, Ndufs1, Ndufs3_predicted, Sdhd |
| GO:0006091 | 47 | 271 | 9.29E-26 | generation of precursor metabolites and energy | Slc25a4, Gapdh, Cox8h, Aldoa, Atp5c1, Atp5g3, Ndufb9_predicted, MGC72942, Cycs, Uqcrb_predicted, Atp5e, Cox6c, Uqcrc1, Ndufb5_predicted, Ndufb4, Sdhb_predicted, Aco2, Ndufs6, Gnas, Cyc1_predicted, Ndufv2, Mor1, Uqcrfs1, Uqcrh, Ech1, Uqcrc2, Atp5j, Ndufs2, Ndufb3_predicted, Idh3g, Ndufs7, Pdha1, Ndufv1, Atp5d, Sdhc, Sdha, Idh3B, Ndufs5b, Pgk1, Tpi1, Pkm2, Ndufs1, Ndufs3_predicted, Pygm, Sdhd, Hk3, Ndufa2_predicted |
| GO:0042773 | 15 | 21 | 9.92E-20 | ATP synthesis coupled electron transport | Cox8h, Ndufb9_predicted, Uqcrc1, Ndufb5_predicted, Ndufb4, Ndufs6, Ndufv2, Ndufs2, Ndufb3_predicted, Ndufs7, Ndufv1, Ndufs5b, Ndufs1, Ndufs3_predicted, Sdhd |
| GO:0042775 | 15 | 21 | 9.92E-20 | ATP synthesis coupled electron transport (sensu Eukaryota) | Cox8h, Ndufb9_predicted, Uqcrc1, Ndufb5_predicted, Ndufb4, Ndufs6, Ndufv2, Ndufs2, Ndufb3_predicted, Ndufs7, Ndufv1, Ndufs5b, Ndufs1, Ndufs3_predicted, Sdhd |
| GO:0006120 | 12 | 18 | 2.03E-15 | mitochondrial electron transport, NADH to ubiquinone | Ndufb9_predicted, Ndufb5_predicted, Ndufb4, Ndufs6, Ndufv2, Ndufs2, Ndufb3_predicted, Ndufs7, Ndufv1, Ndufs5b, Ndufs1, Ndufs3_predicted |
| GO:0006412 | 40 | 356 | 5.87E-15 | protein biosynthesis | Rps29, Rps17, Rpl26, Rps2, Rps11, Rpl19, Rps24, MGC72957, Rps14, Rps27, Rpl9, Rpl10, Rpl37, Rplp1, Rps4x, Rps20, Rpl32, Rplp2, Rps6, Rpl28, Rps12, Rpl17, Rps23, Rpl5, Rpl27a_predicted, Rps19, Rps13, Rpl8, Rps27a, Rpl23, Rpl13, Rpl35, Eef1a2, Rpl34_predicted, Rpl11, Rpl41, Rpl18, Rps5, Eif4a2, Acat1 |
| GO:0009059 | 41 | 405 | 9.40E-14 | macromolecule biosynthesis | Rps29, Rps17, Rpl26, Rps2, Rps11, Rpl19, Rps24, MGC72957, Rps14, Rps27, Rpl9, Rpl10, Rpl37, Rplp1, Rps4x, Rps20, Rpl32, Rplp2, Rps6, Rpl28, Rps12, Rpl17, Rps23, Rpl5, Rpl27a_predicted, Tpi1, Rps19, Rps13, Rpl8, Rps27a, Rpl23,Rpl13, Rpl35, Eef1a2, Rpl34_predicted, Rpl11, Rpl41, Rpl18, Rps5, Eif4a2, Acat1 |
| GO:0006118 | 23 | 129 | 4.14E-13 | electron transport | Cox8h, Ndufb9_predicted, Cycs, Uqcrb_predicted, Cox6c, Uqcrc1, Ndufb5_predicted, Ndufb4, Sdhb_predicted, Ndufs6, Cyc1_predicted, Ndufv2, Uqcrfs1, Uqcrh, Uqcrc2, Ndufs2, Ndufb3_predicted, Ndufs7, Ndufv1, Ndufs5b, Ndufs1, Ndufs3_predicted, Sdhd |
| GO:0009060 | 10 | 17 | 3.10E-12 | aerobic respiration | Atp5g3, Uqcrb_predicted, Sdhb_predicted, Aco2, Mor1, Uqcrh, Idh3g, Sdhc, Sdha, Idh3B |
| GO:0044249 | 49 | 649 | 1.78E-11 | cellular biosynthesis | Rps29, Ckm, MGC72942, Atp5e, Atp5o, Lpl, Rps17, Rpl26, Rps2, Rps11, Rpl19, Rps24, MGC72957, Rps14, Rps27, Rpl9, Rpl10, Rpl37, Rplp1, Rps4x, Rps20, Rpl32, Rplp2, Rps6, Rpl28, Rps12, Rpl17, Atp5d, Rps23, Rpl5, Rpl27a_predicted, Oaz1, Tpi1, Rps19, Rps13, Rpl8, Rps27a, Rpl23, Rpl13, Rpl35, Eef1a2, Rpl34_predicted, Rpl11, Rpl41, Rpl18, Cd74, Rps5, Eif4a2, Acat1 |
| GO:0015980 | 19 | 101 | 1.92E-11 | energy derivation by oxidation of organic compounds | Gapdh, Aldoa, Atp5g3, Uqcrb_predicted, Sdhb_predicted, Aco2, Gnas, Mor1, Uqcrh, Idh3g, Pdha1, Sdhc, Sdha, Idh3B, Pgk1, Tpi1, Pkm2, Pygm, Hk3 |
| GO:0009058 | 52 | 728 | 2.93E-11 | biosynthesis | Fabp3, Rps29, Ckm, MGC72942, Atp5e, Atp5o, Lpl, Rps17, Rpl26, Rps2, Rps11, Rpl19, Rps24, MGC72957, Rps14, Rps27, Rpl9, Rpl10, Rpl37, Rplp1, Rps4x, Rps20, Rpl32, Rplp2, Rps6, Rpl28, Rps12, Rpl17, Atp5d, Rps23, Rpl5, Rpl27a_predicted, Oaz1, Tpi1, Rps19, Rps13, Rpl8, Rps27a, Rpl23, Rpl13, Rpl35, Eef1a2, Rpl34_predicted, Scp2, Rpl11, Cd81, Rpl41, Rpl18, Cd74, Rps5, Eif4a2, Acat1 |
| GO:0006092 | 15 | 62 | 6.36E-11 | main pathways of carbohydrate metabolism | Gapdh, Aldoa, Atp5g3, Sdhb_predicted, Aco2, Mor1, Idh3g, Pdha1, Sdhc, Sdha, Idh3B, Pgk1, Tpi1, Pkm2, Hk3 |
| GO:0045333 | 10 | 22 | 9.16E-11 | cellular respiration | Atp5g3, Uqcrb_predicted, Sdhb_predicted, Aco2, Mor1, Uqcrh, Idh3g, Sdhc, Sdha, Idh3B |
| GO:0015992 | 13 | 48 | 2.71E-10 | proton transport | Atp5b, Atp5c1, Atp5g3, MGC72942, Atp5e, Atp5a1, Atp5o, Atp5h, Atp5f1, Atp5g1, Atp5j, Atp5d, Atp5i |
| GO:0006099 | 8 | 14 | 7.17E-10 | tricarboxylic acid cycle | Atp5g3, Sdhb_predicted, Aco2, Mor1, Idh3g, Sdhc, Sdha, Idh3B |
| GO:0006936 | 18 | 111 | 8.64E-10 | muscle contraction | Actc1, Myh6, Myh7, Tnni3, Tnnt2, Atp2a2, Myl3, Tpm1, Cryab, Aldoa, Hspb6, Actn2_predicted, Pgam2, Gja1, Smpx, Acta1, Casq2, Dmpk_predicted |
| GO:0006818 | 13 | 53 | 1.05E-09 | hydrogen transport | Atp5b, Atp5c1, Atp5g3, MGC72942, Atp5e, Atp5a1, Atp5o, Atp5h, Atp5f1, Atp5g1, Atp5j, Atp5d, Atp5i |
| GO:0046356 | 8 | 15 | 1.50E-09 | acetyl-CoA catabolism | Atp5g3, Sdhb_predicted, Aco2, Mor1, Idh3g, Sdhc, Sdha, Idh3B |
| GO:0044237 | 134 | 3460 | 2.48E-09 | cellular metabolism | Actc1, Myh7, Fabp3, Slc25a4, Cryab, Rps29, Gapdh, Cox8h, Aldoa, Atp5c1, Atp5g3, Ndufb9_predicted, Ckm, Hspb6, MGC72942, Cycs, Uqcrb_predicted, Atp5e, Ankrd1, Gpx1, Atp5o, Phyh, Ubb, Ybx1, Hspb7, Cox6c, Uqcrc1, Lpl, Acadl, Ndufb5_predicted, Ndufb4, Sdhb_predicted, Aco2, Ndufs6, Rps17, Rpl26, Rps2, Gnas, Rps11, Hspa8, Cyc1_predicted, Acadm, Rpl19, Rps24, Fhl2, MGC72957, Rps14, Ndufv2, Mor1, Cd36, Hadhb, Acaa2, Rps27, Uqcrfs1, Uqcrh, Tmsb4x, Rpl9, Ubc, Rpl10, Rpl37, Rplp1, Ech1, Pam, Rps4x, Rps20, Uqcrc2, Atp5j, Rpl32, Rplp2, Acta1, Ndufs2, Ndufb3_predicted, Rps6, Idh3g, Ndufs7, Rpl28, Pdha1, Rps12, Prkar1a, Rpl17, Ndufv1, Gsn, Atp5d, Sdhc, Rps23, Rpl5, Rpl27a_predicted, Oaz1, Sdha, Got2, Idh3B, Hspcb, Ndufs5b, Pgk1, Maoa, Tpi1, Sod1, Pkm2, Etfb, Ndufs1, Pink1_predicted, Gpx4, Ndufs3_predicted, Pygm, Rps19, Rps13, Rpl8, Rps27a, Rpl23, Rpl13, Rpl35, Eef1a2,Rpl34_predicted, Sdhd, Scp2, Hspe1, RGD1305801_predicted, Rpl11, Acsl1, Dmpk_predicted, Prdx2, Nudt4, Hspd1, Cd81, Rpl41, Nme2, Rpl18, Hk3, H3f3b, Cd74, Rps5, Eif4a2, Ndufa2_predicted, Acat1 |
| GO:0009109 | 8 | 16 | 2.93E-09 | coenzyme catabolism | Atp5g3, Sdhb_predicted, Aco2, Mor1, Idh3g, Sdhc, Sdha, Idh3B |
| GO:0006732 | 16 | 94 | 3.77E-09 | coenzyme metabolism | Atp5g3, MGC72942, Atp5e, Atp5o, Sdhb_predicted, Aco2, Mor1, Idh3g, Pdha1, Atp5d, Sdhc, Sdha, Idh3B, Tpi1, Ndufs1, Scp2 |
| GO:0006084 | 9 | 23 | 4.23E-09 | acetyl-CoA metabolism | Atp5g3, Sdhb_predicted, Aco2, Mor1, Idh3g, Pdha1, Sdhc, Sdha, Idh3B |
| GO:0008152 | 138 | 3675 | 9.51E-09 | metabolism | Actc1, Myh7, Fabp3, Slc25a4, Cryab, Rps29, Gapdh, Cox8h, Aldoa, Atp5c1, Atp5g3, Ndufb9_predicted, Ckm, Hspb6, MGC72942, Cycs, Uqcrb_predicted, Atp5e, Ankrd1, Gpx1, Atp5o, Phyh, Ubb, Ybx1, Hspb7, Cox6c, Uqcrc1, Lpl, Acadl, Ndufb5_predicted, Ndufb4, Sdhb_predicted, Aco2, Actn2_predicted, Ndufs6, Rps17, Rpl26, Rps2, Gnas, Rps11, Hspa8, Cyc1_predicted, Acadm, Rpl19, Rps24, Fhl2, MGC72957, Rps14, Ndufv2, Mor1, Cd36, Hadhb, Clu, Acaa2, Rps27, Uqcrfs1, Uqcrh, Tmsb4x, Rpl9, Ubc, Rpl10, Rpl37, Rplp1, Gja1, Ech1, Pam, Rps4x, Rps20, Uqcrc2, Atp5j, Rpl32, Rplp2, Acta1, Ndufs2, Ndufb3_predicted, Rps6, Idh3g, Ndufs7, Rpl28, Pdha1, Rps12, Prkar1a, Rpl17, Ndufv1, Gsn, Atp5d, Sdhc, Rps23, Rpl5, Rpl27a_predicted, Oaz1, Sdha, Got2, Idh3B, Hspcb, Ndufs5b, Pgk1, Maoa, Tpi1, Sod1, Pkm2, Etfb, Ndufs1, Pink1_predicted, Gpx4, Ndufs3_predicted, Pygm, Rps19, Rps13, Rpl8, Rps27a, Rpl23, Hadhsc, Rpl13, Rpl35, Eef1a2, Rpl34_predicted, Sdhd, Scp2, Hspe1, RGD1305801_predicted, Rpl11, Acsl1, Dmpk_predicted, Prdx2, Nudt4, Hspd1, Cd81, Rpl41, Nme2, Rpl18, Hk3, H3f3b, Cd74, Rps5, Eif4a2, Ndufa2_predicted, Acat1 |
| GO:0051187 | 8 | 20 | 2.61E-08 | cofactor catabolism | Atp5g3, Sdhb_predicted, Aco2, Mor1, Idh3g, Sdhc, Sdha, Idh3B |
| GO:0051186 | 16 | 117 | 9.53E-08 | cofactor metabolism | Atp5g3, MGC72942, Atp5e, Atp5o, Sdhb_predicted, Aco2, Mor1, Idh3g, Pdha1, Atp5d, Sdhc, Sdha, Idh3B, Tpi1, Ndufs1, Scp2 |
| GO:0006096 | 7 | 18 | 2.56E-07 | glycolysis | Gapdh, Aldoa, Pdha1, Pgk1, Tpi1, Pkm2, Hk3 |
| GO:0016310 | 28 | 355 | 3.18E-07 | phosphorylation | Cox8h, Atp5c1, Ndufb9_predicted, MGC72942, Uqcrb_predicted, Atp5e, Uqcrc1, Ndufb5_predicted, Ndufb4, Ndufs6, Cyc1_predicted, Ndufv2, Uqcrh, Ndufs2, Ndufb3_predicted, Ndufs7, Prkar1a, Ndufv1, Atp5d, Ndufs5b, Pgk1, Ndufs1, Pink1_predicted, Ndufs3_predicted, Sdhd, RGD1305801_predicted, Dmpk_predicted, Cd81 |
| GO:0006941 | 6 | 16 | 2.49E-06 | striated muscle contraction | Myh6, Myh7, Aldoa, Pgam2, Smpx, Casq2 |
| GO:0006007 | 7 | 26 | 4.40E-06 | glucose catabolism | Gapdh, Aldoa, Pdha1, Pgk1, Tpi1, Pkm2, Hk3 |
| GO:0044260 | 62 | 1362 | 1.17E-05 | cellular macromolecule metabolism | Actc1, Myh7, Cryab, Rps29, Hspb6, Ubb, Hspb7, Rps17, Rpl26, Rps2, Rps11, Hspa8, Rpl19, Rps24, MGC72957, Rps14, Rps27, Tmsb4x, Rpl9, Ubc, Rpl10, Rpl37, Rplp1, Pam, Rps4x, Rps20, Rpl32, Rplp2, Acta1, Rps6, Rpl28, Rps12, Prkar1a, Rpl17, Gsn, Rps23, Rpl5, Rpl27a_predicted, Hspcb, Pink1_predicted, Pygm, Rps19, Rps13, Rpl8, Rps27a, Rpl23, Rpl13, Rpl35, Eef1a2, Rpl34_predicted, Hspe1, RGD1305801_predicted, Rpl11, Dmpk_predicted, Hspd1, Cd81, Rpl41, Rpl18, Cd74, Rps5, Eif4a2, Acat1 |
| GO:0044267 | 61 | 1334 | 1.23E-05 | cellular protein metabolism | Actc1, Myh7, Cryab, Rps29, Hspb6, Ubb, Hspb7, Rps17, Rpl26, Rps2, Rps11, Hspa8, Rpl19, Rps24, MGC72957, Rps14, Rps27, Tmsb4x, Rpl9, Ubc, Rpl10, Rpl37, Rplp1, Pam, Rps4x, Rps20, Rpl32, Rplp2, Acta1, Rps6, Rpl28, Rps12, Prkar1a, Rpl17, Gsn, Rps23, Rpl5, Rpl27a_predicted, Hspcb, Pink1_predicted, Rps19, Rps13, Rpl8, Rps27a, Rpl23, Rpl13, Rpl35, Eef1a2, Rpl34_predicted, Hspe1, RGD1305801_predicted, Rpl11, Dmpk_predicted, Hspd1, Cd81, Rpl41, Rpl18, Cd74, Rps5, Eif4a2, Acat1 |
| GO:0019320 | 7 | 30 | 1.24E-05 | hexose catabolism | Gapdh, Aldoa, Pdha1, Pgk1, Tpi1, Pkm2, Hk3 |
| GO:0046365 | 7 | 30 | 1.24E-05 | monosaccharide catabolism | Gapdh, Aldoa, Pdha1, Pgk1, Tpi1, Pkm2, Hk3 |
| GO:0046164 | 7 | 30 | 1.24E-05 | alcohol catabolism | Gapdh, Aldoa, Pdha1, Pgk1, Tpi1, Pkm2, Hk3 |
| GO:0006631 | 14 | 136 | 1.89E-05 | fatty acid metabolism | Fabp3, Phyh, Lpl, Acadl, Acadm, Cd36, Hadhb, Acaa2, Ech1, Tpi1, Etfb, Scp2, Acsl1, Cd74 |
| GO:0006793 | 28 | 440 | 2.14E-05 | phosphorus metabolism | Cox8h, Atp5c1, Ndufb9_predicted, MGC72942, Uqcrb_predicted, Atp5e, Uqcrc1, Ndufb5_predicted, Ndufb4, Ndufs6, Cyc1_predicted, Ndufv2, Uqcrh, Ndufs2, Ndufb3_predicted, Ndufs7, Prkar1a, Ndufv1, Atp5d, Ndufs5b, Pgk1, Ndufs1, Pink1_predicted, Ndufs3_predicted, Sdhd, RGD1305801_predicted, Dmpk_predicted, Cd81 |
| GO:0006796 | 28 | 440 | 2.14E-05 | phosphate metabolism | Cox8h, Atp5c1, Ndufb9_predicted, MGC72942, Uqcrb_predicted, Atp5e, Uqcrc1, Ndufb5_predicted, Ndufb4, Ndufs6, Cyc1_predicted, Ndufv2, Uqcrh, Ndufs2, Ndufb3_predicted, Ndufs7, Prkar1a, Ndufv1, Atp5d, Ndufs5b, Pgk1, Ndufs1, Pink1_predicted, Ndufs3_predicted, Sdhd, RGD1305801_predicted, Dmpk_predicted, Cd81 |
| GO:0044275 | 8 | 48 | 4.05E-05 | cellular carbohydrate catabolism | Gapdh, Aldoa, Pdha1, Pgk1, Tpi1, Pkm2, Pygm, Hk3 |
| GO:0019538 | 64 | 1478 | 4.09E-05 | protein metabolism | Actc1, Myh7, Cryab, Rps29, Hspb6, Ubb, Hspb7, Actn2_predicted, Rps17, Rpl26, Rps2, Rps11, Hspa8, Rpl19, Rps24, MGC72957, Rps14, Rps27, Tmsb4x, Rpl9, Ubc, Rpl10, Rpl37, Rplp1, Gja1, Pam, Rps4x, Rps20, Rpl32, Rplp2, Acta1, Rps6, Rpl28, Rps12, Prkar1a, Rpl17, Gsn, Rps23, Rpl5, Rpl27a_predicted, Hspcb, Pink1_predicted, Rps19, Rps13, Rpl8, Rps27a, Rpl23, Rpl13, Rpl35, Eef1a2, Rpl34_predicted, Hspe1, RGD1305801_predicted, Rpl11, Dmpk_predicted, Hspd1, Cd81, Rpl41, Rpl18, H3f3b, Cd74, Rps5, Eif4a2, Acat1 |
| GO:0016052 | 8 | 49 | 4.72E-05 | carbohydrate catabolism | Gapdh, Aldoa, Pdha1, Pgk1, Tpi1, Pkm2, Pygm, Hk3 |
| GO:0043170 | 82 | 2075 | 5.67E-05 | macromolecule metabolism | Actc1, Myh7, Cryab, Rps29, Gapdh, Aldoa, Atp5g3, Hspb6, Ubb, Hspb7, Sdhb_predicted, Aco2, Actn2_predicted, Rps17, Rpl26, Rps2, Rps11, Hspa8, Rpl19, Rps24, MGC72957, Rps14, Mor1, Rps27, Tmsb4x, Rpl9, Ubc, Rpl10, Rpl37, Rplp1, Gja1, Pam, Rps4x, Rps20, Rpl32, Rplp2, Acta1, Rps6, Idh3g, Rpl28, Pdha1, Rps12, Prkar1a, Rpl17, Gsn, Sdhc, Rps23, Rpl5, Rpl27a_predicted, Sdha, Idh3B, Hspcb, Pgk1, Tpi1, Sod1, Pkm2, Pink1_predicted, Gpx4, Pygm, Rps19, Rps13, Rpl8, Rps27a, Rpl23, Rpl13, Rpl35, Eef1a2, Rpl34_predicted, Hspe1, RGD1305801_predicted, Rpl11, Dmpk_predicted, Hspd1, Cd81, Rpl41, Rpl18, Hk3, H3f3b, Cd74, Rps5, Eif4a2, Acat1 |
| GO:0044262 | 16 | 193 | 7.12E-05 | cellular carbohydrate metabolism | Gapdh, Aldoa, Atp5g3, Sdhb_predicted, Aco2, Mor1, Idh3g, Pdha1, Sdhc, Sdha, Idh3B, Pgk1, Tpi1, Pkm2, Pygm, Hk3 |
| GO:0019395 | 6 | 30 | 0.000134298 | fatty acid oxidation | Fabp3, Phyh, Acadm, Hadhb, Ech1, Etfb |
| GO:0008016 | 6 | 31 | 0.000162774 | regulation of heart contraction | Actc1, Tnni3, Tpm1, Hspb7, Gja1, Dmpk_predicted |
| GO:0015672 | 15 | 186 | 0.00016415 | monovalent inorganic cation transport | Atp5b, Atp5c1, Atp5g3, MGC72942, Atp5e, Atp5a1, Atp5o, Atp5h, Atp5f1, Atp5g1, Atp5j, Ndufa9, Atp5d, Atp5i, Kcnip2 |
| GO:0030239 | 3 | 5 | 0.00019467 | myofibril assembly | Ttn, Acta1, Actg1 |
| GO:0006635 | 5 | 21 | 0.000208818 | fatty acid beta-oxidation | Fabp3, Acadm, Hadhb, Ech1, Etfb |
| GO:0044248 | 20 | 305 | 0.000238592 | cellular catabolism | Gapdh, Aldoa, Atp5g3, Sdhb_predicted, Aco2, Mor1, Ubc, Idh3g, Pdha1, Sdhc, Sdha, Got2, Idh3B, Pgk1, Maoa, Tpi1, Sod1, Pkm2, Pygm, Hk3 |
| GO:0009145 | 4 | 14 | 0.000441216 | purine nucleoside triphosphate biosynthesis | MGC72942, Atp5e, Atp5o, Atp5d |
| GO:0009201 | 4 | 14 | 0.000441216 | ribonucleoside triphosphate biosynthesis | MGC72942, Atp5e, Atp5o, Atp5d |
| GO:0009206 | 4 | 14 | 0.000441216 | purine ribonucleoside triphosphate biosynthesis | MGC72942, Atp5e, Atp5o, Atp5d |
| GO:0006753 | 4 | 14 | 0.000441216 | nucleoside phosphate metabolism | MGC72942, Atp5e, Atp5o, Atp5d |
| GO:0006754 | 4 | 14 | 0.000441216 | ATP biosynthesis | MGC72942, Atp5e, Atp5o, Atp5d |
| GO:0006986 | 6 | 37 | 0.000448315 | response to unfolded protein | Hspb6, Hspb7, Hspa8, Hspcb, Hspe1, Hspd1 |
| GO:0051789 | 6 | 37 | 0.000448315 | response to protein stimulus | Hspb6, Hspb7, Hspa8, Hspcb, Hspe1, Hspd1 |
| GO:0015985 | 3 | 7 | 0.000654131 | energy coupled proton transport, down electrochemical gradient | MGC72942, Atp5e, Atp5d |
| GO:0015986 | 3 | 7 | 0.000654131 | ATP synthesis coupled proton transport | MGC72942, Atp5e, Atp5d |
| GO:0045844 | 2 | 2 | 0.000747565 | positive regulation of striated muscle development | Csrp3, Gja1 |
| GO:0009056 | 21 | 360 | 0.000799903 | catabolism | Gapdh, Aldoa, Atp5g3, Lpl, Sdhb_predicted, Aco2, Mor1, Ubc, Idh3g, Pdha1, Sdhc, Sdha, Got2, Idh3B, Pgk1, Maoa, Tpi1, Sod1, Pkm2, Pygm, Hk3 |
| GO:0009199 | 4 | 17 | 0.000983492 | ribonucleoside triphosphate metabolism | MGC72942, Atp5e, Atp5o, Atp5d |
| GO:0009142 | 4 | 17 | 0.000983492 | nucleoside triphosphate biosynthesis | MGC72942, Atp5e, Atp5o, Atp5d |
| GO:0009205 | 4 | 17 | 0.000983492 | purine ribonucleoside triphosphate metabolism | MGC72942, Atp5e, Atp5o, Atp5d |
| GO:0046034 | 4 | 17 | 0.000983492 | ATP metabolism | MGC72942, Atp5e, Atp5o, Atp5d |
| GO:0005975 | 16 | 246 | 0.001112274 | carbohydrate metabolism | Gapdh, Aldoa, Atp5g3, Sdhb_predicted, Aco2, Mor1, Idh3g, Pdha1, Sdhc, Sdha, Idh3B, Pgk1, Tpi1, Pkm2, Pygm, Hk3 |
| GO:0006937 | 5 | 30 | 0.001197049 | regulation of muscle contraction | Tnni3, Atp2a2, Tpm1, Hspb6, Casq2 |
| GO:0009144 | 4 | 18 | 0.001237629 | purine nucleoside triphosphate metabolism | MGC72942, Atp5e, Atp5o, Atp5d |
| GO:0007517 | 10 | 116 | 0.001237751 | muscle development | Actc1, Myh6, Myh7, Cryab, Csrp3, Ttn, Gja1, Des, Acta1, Actg1 |
| GO:0006812 | 18 | 307 | 0.001801657 | cation transport | Atp2a2, Atp5b, Pln, Atp5c1, Atp5g3, MGC72942, Atp5e, Atp5a1, Atp5o, Atp5h, Atp5f1, Atp5g1, Vdac1, Atp5j, Ndufa9, Atp5d, Atp5i, Kcnip2 |
| GO:0009152 | 4 | 20 | 0.001877317 | purine ribonucleotide biosynthesis | MGC72942, Atp5e, Atp5o, Atp5d |
| GO:0006810 | 59 | 1523 | 0.001965657 | transport | Hba-a1, Atp2a2, Slc25a4, Slc25a3, Cox8h, Atp5b, Pln, Atp5c1, Atp5g3, Ndufb9_predicted, MGC72942, Cycs, Uqcrb_predicted, Atp5e, Atp5a1, Atp5o, Atp5h, Cox6c, Atp5f1, Uqcrc1, Atp5g1, Ndufb5_predicted, Ndufb4, Hbb, Sdhb_predicted, Ndufs6, Vdac3, Cyc1_predicted, Ndufv2, Cd36, Uqcrfs1, Uqcrh, Vdac1, Gja1, Uqcrc2, Vdac2, Atp5j, Ndufa9, Ndufs2, Ndufb3_predicted, Ndufs7, Ndufv1, Gsn, Atp5d, Got2, Ndufs5b, Maoa, Ndufs1, Ndufs3_predicted, Atp5i, Rpl23, Sdhd, Scp2, Nudt4, Hspd1, Kcnip2, Cd74, Cd63, Tuba4 |
| GO:0048628 | 3 | 10 | 0.002110143 | myoblast maturation | Ttn, Acta1, Actg1 |
| GO:0006006 | 7 | 67 | 0.002252798 | glucose metabolism | Gapdh, Aldoa, Pdha1, Pgk1, Tpi1, Pkm2, Hk3 |
| GO:0009141 | 4 | 22 | 0.0027156 | nucleoside triphosphate metabolism | MGC72942, Atp5e, Atp5o, Atp5d |
| GO:0048627 | 3 | 11 | 0.002843255 | myoblast development | Ttn, Acta1, Actg1 |
| GO:0006164 | 4 | 24 | 0.003779744 | purine nucleotide biosynthesis | MGC72942, Atp5e, Atp5o, Atp5d |
| GO:0006457 | 8 | 93 | 0.003825908 | protein folding | Cryab, Hspb6, Hspb7, Hspa8, Hspcb, Hspe1, Hspd1, Cd74 |
| GO:0044238 | 110 | 3345 | 0.003997872 | primary metabolism | Actc1, Myh7, Fabp3, Cryab, Rps29, Gapdh, Aldoa, Atp5g3, Ckm, Hspb6, MGC72942, Atp5e, Ankrd1, Atp5o, Phyh, Ubb, Ybx1, Hspb7, Lpl, Acadl, Sdhb_predicted, Aco2, Actn2_predicted, Rps17, Rpl26, Rps2, Rps11, Hspa8, Acadm, Rpl19, Rps24, Fhl2, MGC72957, Rps14, Mor1, Cd36, Hadhb, Clu, Acaa2, Rps27, Tmsb4x, Rpl9, Ubc, Rpl10, Rpl37, Rplp1, Gja1, Ech1, Pam, Rps4x, Rps20, Rpl32, Rplp2, Acta1, Rps6, Idh3g, Rpl28, Pdha1, Rps12, Prkar1a, Rpl17, Gsn, Atp5d, Sdhc, Rps23, Rpl5, Rpl27a_predicted, Oaz1, Sdha, Got2, Idh3B, Hspcb, Pgk1, Maoa, Tpi1, Sod1, Pkm2, Etfb, Ndufs1, Pink1_predicted, Gpx4, Pygm, Rps19, Rps13, Rpl8, Rps27a, Rpl23, Hadhsc, Rpl13, Rpl35, Eef1a2, Rpl34_predicted, Scp2, Hspe1, RGD1305801_predicted, Rpl11, Acsl1, Dmpk_predicted, Nudt4, Hspd1, Cd81, Rpl41, Nme2, Rpl18, Hk3, H3f3b, Cd74, Rps5, Eif4a2, Acat1 |
| GO:0045214 | 2 | 4 | 0.0043248 | sarcomere organization | Ttn, Actg1 |
| GO:0009260 | 4 | 25 | 0.0044047 | ribonucleotide biosynthesis | MGC72942, Atp5e, Atp5o, Atp5d |
| GO:0009150 | 4 | 26 | 0.005095756 | purine ribonucleotide metabolism | MGC72942, Atp5e, Atp5o, Atp5d |
| GO:0006752 | 4 | 26 | 0.005095756 | group transfer coenzyme metabolism | MGC72942, Atp5e, Atp5o, Atp5d |
| GO:0015669 | 2 | 5 | 0.007078221 | gas transport | Hba-a1, Hbb |
| GO:0015671 | 2 | 5 | 0.007078221 | oxygen transport | Hba-a1, Hbb |
| GO:0019752 | 18 | 361 | 0.009816502 | carboxylic acid metabolism | Fabp3, Atp5g3, Phyh, Lpl, Acadl, Aco2, Acadm, Cd36, Hadhb, Acaa2, Ech1, Got2, Tpi1, Etfb, Scp2, Acsl1, Cd81, Cd74 |
| GO:0009117 | 8 | 109 | 0.009862066 | nucleotide metabolism | MGC72942, Atp5e, Atp5o, Atp5d, Tpi1, Ndufs1, Nudt4, Nme2 |
