# Supplementary material for: Gene expression profile of rat left ventricles reveals persisting changes following chronic mild exercise protocol: implications for cardioprotection
Source: BMC Genomics. 2009 Jul 30;10:342. doi: 10.1186/1471-2164-10-342 (PMC2907697; doi:10.1186/1471-2164-10-342)
Supplement: Additional file 4 — Gene ontology (GO) analysis for molecular functions. In this table, the gene ontology analysis according to molecular functions of the 300 most expressed genes in the overall animal population is reported. The genes associated with each molecular function among the 300 most expressed genes are reported. [file 1471-2164-10-342-S4.doc]

**GENE ONTOLOGY (GO) ANALYSIS FOR MOLECULAR FUNCTIONS.**

**n=number of genes associated with the molecular function among the 300 most expressed genes.**

**N=number of genes associated with the molecular function present in the GeneChip Rat Genome 230 v2.0 Array.**

**P value = Fisher exact test p value**

| **GO ID** | **n** | **N** | **P value** | **GO Names** | **Gene Symbol** |
| --- | --- | --- | --- | --- | --- |
| GO:0003735 | 45 | 100 | 2.07E-44 | structural constituent of ribosome | Rps29, Rps17, Rps3a, Rps2, Rps11, Rpl19, Rps24, MGC72957, Rps14, Rps27, Rpl9, Rpl10, Rpl37, Rplp1, Rps4x, Rps20, Rpl31, Rpl32, Rplp2, Rps6, Rpl28, Rpl27, Rps12, Rpl17, Rps23, Rpl5, Rpl30, Rpl27a_predicted, Rpl7, Rps8, Rps15a, Rps19, Rps13, Rps16, Rpl8, Rpl23, Rpl13, Rpl35, Rpl34_predicted, Rpl35a, Rpl11, Rpl41, Rpl18, Rps26, Rps5 |
| GO:0005198 | 60 | 338 | 7.31E-33 | structural molecule activity | Actc1, Myh7, Tnnt2, Myl3, Tpm1, Cryab, Rps29, Actn2_predicted, Rps17, Rps3a, Rps2, Rps11, Rpl19, Rps24, MGC72957, Rps14, Rps27, Rpl9, Rpl10, Dsp, Rpl37, Rplp1, Rps4x, Rps20, Rpl31, Rpl32, Rplp2, Des, Acta1, Rps6, Rpl28, Rpl27, Rps12, Actg1, Rpl17, Gsn, Mgp, Rps23, Rpl5, Rpl30, Rpl27a_predicted, Rpl7, Rps8, Hrc, Rps15a, Rps19, Rps13, Rps16, Rpl8, Rpl23, Rpl13, Rpl35, Rpl34_predicted, Rpl35a, Rpl11, Rpl41, Rpl18, Rps26, Rps5, Tuba4 |
| GO:0008137 | 18 | 24 | 7.18E-24 | NADH dehydrogenase (ubiquinone) activity | Ndufb9_predicted, Ndufb5_predicted, Ndufb4, Ndufs6, Ndufa12_predicted, Ndufab1_predicted, Ndufv2, Ndufa9, Ndufs2, Ndufb3_predicted, Ndufv1, Ndufs5b, Ndufs1, Ndufs3_predicted, Ndufa8, Ndufa7_predicted, Ndufa6_predicted, Ndufa2_predicted |
| GO:0050136 | 18 | 24 | 7.18E-24 | NADH dehydrogenase (quinone) activity | Ndufb9_predicted, Ndufb5_predicted, Ndufb4, Ndufs6, Ndufa12_predicted, Ndufab1_predicted, Ndufv2, Ndufa9, Ndufs2, Ndufb3_predicted, Ndufv1, Ndufs5b, Ndufs1, Ndufs3_predicted, Ndufa8, Ndufa7_predicted, Ndufa6_predicted, Ndufa2_predicted |
| GO:0003954 | 18 | 24 | 7.18E-24 | NADH dehydrogenase activity | Ndufb9_predicted, Ndufb5_predicted, Ndufb4, Ndufs6, Ndufa12_predicted, Ndufab1_predicted, Ndufv2, Ndufa9, Ndufs2, Ndufb3_predicted, Ndufv1, Ndufs5b, Ndufs1, Ndufs3_predicted, Ndufa8, Ndufa7_predicted, Ndufa6_predicted, Ndufa2_predicted |
| GO:0016491 | 57 | 478 | 6.15E-22 | oxidoreductase activity | Cox6a2, Gapdh, Ldhb, Cox8h, Cox4i1, Fth1, Atp5g3, Ndufb9_predicted, Cox7a2, Cycs, Gpx1, Phyh, Mdh1, Cox6c, Acadl, Cox5a, Cox5b, Ndufb5_predicted, Ndufb4, Cox7b, Sdhb_predicted, Ndufs6, Ndufa12_predicted, Cyc1_predicted, Acadm, Ndufab1_predicted, Ndufv2, Mor1, Hadhb, Acaa2, Uqcrfs1, Uqcrh, Ldha, Pam, Uqcrc2, Ndufa9, Ndufs2, Ndufb3_predicted, Idh3g, Pdha1, Ndufv1, Sdhc, Sdha, Idh3B, Ndufs5b, Maoa, Sod1, Ndufs1, Gpx4, Ndufs3_predicted, Hadhsc, Sdhd, Ndufa8, Ndufa7_predicted, Prdx2, Ndufa6_predicted, Ndufa2_predicted |
| GO:0016655 | 18 | 28 | 6.34E-22 | oxidoreductase activity, acting on NADH or NADPH, quinone or similar compound as acceptor | Ndufb9_predicted, Ndufb5_predicted, Ndufb4, Ndufs6, Ndufa12_predicted, Ndufab1_predicted, Ndufv2, Ndufa9, Ndufs2, Ndufb3_predicted, Ndufv1, Ndufs5b, Ndufs1, Ndufs3_predicted, Ndufa8, Ndufa7_predicted, Ndufa6_predicted, Ndufa2_predicted |
| GO:0016651 | 18 | 42 | 1.21E-17 | oxidoreductase activity, acting on NADH or NADPH | Ndufb9_predicted, Ndufb5_predicted, Ndufb4, Ndufs6, Ndufa12_predicted, Ndufab1_predicted, Ndufv2, Ndufa9, Ndufs2, Ndufb3_predicted, Ndufv1, Ndufs5b, Ndufs1, Ndufs3_predicted, Ndufa8, Ndufa7_predicted, Ndufa6_predicted, Ndufa2_predicted |
| GO:0009055 | 27 | 150 | 5.73E-15 | electron carrier activity | Ndufb9_predicted, Cycs, Phyh, Cox5a, Ndufb5_predicted, Ndufb4, Ndufs6, Ndufa12_predicted, Cyc1_predicted, Ndufab1_predicted, Ndufv2, Pam, Ndufa9, Ndufs2, Ndufb3_predicted, Ndufv1, Sdhc, Sdha, Ndufs5b, Ndufs1, Gpx4, Ndufs3_predicted, Ndufa8, Ndufa7_predicted, Prdx2, Ndufa6_predicted, Ndufa2_predicted |
| GO:0003723 | 33 | 277 | 1.03E-12 | RNA binding | Rps29, Ybx1, Rpl26, Rps2, Rps11, Rpl19, Rps24, Rps14, Rps27, Rpl9, Rpl37, Rplp1, Rps4x, Rps20, Rpl32, Rplp2, Rps6, Rpl28, Rps12, Rpl17, Rps23, Rpl5, Rpl27a_predicted, Rpl7, Rps19, Rps13, Rpl8, Rpl13, Rpl34_predicted, Rpl35a, Rpl11, Rpl18, Rps5 |
| GO:0015078 | 16 | 59 | 3.45E-12 | hydrogen ion transporter activity | Cox6a2, Cox8h, Cox4i1, Atp5g3, MGC72942, Atp5e, Atp5o, Atp5h, Cox6c, Cox5a, Cox5b, Cox7b, Uqcrfs1, Uqcrh, Uqcrc2, Atp5d |
| GO:0015077 | 16 | 65 | 1.75E-11 | monovalent inorganic cation transporter activity | Cox6a2, Cox8h, Cox4i1, Atp5g3, MGC72942, Atp5e, Atp5o, Atp5h, Cox6c, Cox5a, Cox5b, Cox7b, Uqcrfs1, Uqcrh, Uqcrc2, Atp5d |
| GO:0015002 | 7 | 17 | 1.99E-07 | heme-copper terminal oxidase activity | Cox6a2, Cox8h, Cox4i1, Cox6c, Cox5a, Cox5b, Cox7b |
| GO:0016675 | 7 | 17 | 1.99E-07 | oxidoreductase activity, acting on heme group of donors | Cox6a2, Cox8h, Cox4i1, Cox6c, Cox5a, Cox5b, Cox7b |
| GO:0016676 | 7 | 17 | 1.99E-07 | oxidoreductase activity, acting on heme group of donors, oxygen as acceptor | Cox6a2, Cox8h, Cox4i1, Cox6c, Cox5a, Cox5b, Cox7b |
| GO:0004129 | 7 | 17 | 1.99E-07 | cytochrome-c oxidase activity | Cox6a2, Cox8h, Cox4i1, Cox6c, Cox5a, Cox5b, Cox7b |
| GO:0019843 | 5 | 7 | 3.45E-07 | rRNA binding | Rps11, Rpl37, Rpl5, Rpl8, Rpl11 |
| GO:0008177 | 3 | 3 | 2.23E-05 | succinate dehydrogenase (ubiquinone) activity | Sdhb_predicted, Sdha, Sdhd |
| GO:0008121 | 3 | 3 | 2.23E-05 | ubiquinol-cytochrome-c reductase activity | Uqcrfs1, Uqcrh, Uqcrc2 |
| GO:0016681 | 3 | 3 | 2.23E-05 | oxidoreductase activity, acting on diphenols and related substances as donors, cytochrome as acceptor | Uqcrfs1, Uqcrh, Uqcrc2 |
| GO:0016679 | 3 | 3 | 2.23E-05 | oxidoreductase activity, acting on diphenols and related substances as donors | Uqcrfs1, Uqcrh, Uqcrc2 |
| GO:0016635 | 3 | 3 | 2.23E-05 | oxidoreductase activity, acting on the CH-CH group of donors, quinone or related compound as acceptor | Sdhb_predicted, Sdha, Sdhd |
| GO:0015482 | 3 | 3 | 2.23E-05 | voltage-gated anion channel porin activity | Vdac3, Vdac1, Vdac2 |
| GO:0016614 | 10 | 85 | 0.000128458 | oxidoreductase activity, acting on CH-OH group of donors | Ldhb, Atp5g3, Mdh1, Mor1, Hadhb, Acaa2, Ldha, Idh3g, Idh3B, Hadhsc |
| GO:0008307 | 5 | 20 | 0.000188251 | structural constituent of muscle | Actc1, Myh7, Myl3, Tpm1, Actn2_predicted |
| GO:0004448 | 3 | 5 | 0.000213375 | isocitrate dehydrogenase activity | Atp5g3, Idh3g, Idh3B |
| GO:0015288 | 3 | 5 | 0.000213375 | porin activity | Vdac3, Vdac1, Vdac2 |
| GO:0016616 | 9 | 77 | 0.000294199 | oxidoreductase activity, acting on the CH-OH group of donors, NAD or NADP as acceptor | Ldhb, Atp5g3, Mor1, Hadhb, Acaa2, Ldha, Idh3g, Idh3B, Hadhsc |
| GO:0005506 | 9 | 79 | 0.000357491 | iron ion binding | Hba-a1, Mb, Cycs, Phyh, Cox5a, Hbb, Aco2, Ndufv2, Uqcrfs1 |
| GO:0005344 | 3 | 6 | 0.000417858 | oxygen transporter activity | Hba-a1, Mb, Hbb |
| GO:0051540 | 4 | 15 | 0.000661986 | metal cluster binding | Sdhb_predicted, Aco2, Ndufv2, Uqcrfs1 |
| GO:0051536 | 4 | 15 | 0.000661986 | iron-sulfur cluster binding | Sdhb_predicted, Aco2, Ndufv2, Uqcrfs1 |
| GO:0051537 | 3 | 7 | 0.000716035 | 2 iron, 2 sulfur cluster binding | Sdhb_predicted, Ndufv2, Uqcrfs1 |
| GO:0016408 | 3 | 8 | 0.001121847 | C-acyltransferase activity | Hadhb, Acaa2, Acat1 |
| GO:0008553 | 4 | 20 | 0.002103125 | hydrogen-exporting ATPase activity, phosphorylative mechanism | Atp5g3, MGC72942, Atp5e, Atp5o |
| GO:0016627 | 5 | 33 | 0.002138044 | oxidoreductase activity, acting on the CH-CH group of donors | Acadl, Sdhb_predicted, Acadm, Sdha, Sdhd |
| GO:0003988 | 2 | 3 | 0.002340808 | acetyl-CoA C-acyltransferase activity | Hadhb, Acaa2 |
| GO:0004459 | 2 | 3 | 0.002340808 | L-lactate dehydrogenase activity | Ldhb, Ldha |
| GO:0004449 | 2 | 3 | 0.002340808 | isocitrate dehydrogenase (NAD+) activity | Idh3g, Idh3B |
| GO:0015075 | 24 | 472 | 0.003487597 | ion transporter activity | Atp2a2, Cox6a2, Slc25a3, Cox8h, Cox4i1, Atp5g3, MGC72942, Atp5e, Atp5o, Atp5h, Cox6c, Cox5a, Cox5b, Cox7b, Vdac3, Uqcrfs1, Uqcrh, Ryr2, Vdac1, Gja1, Uqcrc2, Vdac2, Atp5d, Kcnip2 |
| GO:0003985 | 2 | 4 | 0.004594447 | acetyl-CoA C-acetyltransferase activity | Hadhb, Acat1 |
| GO:0004457 | 2 | 4 | 0.004594447 | lactate dehydrogenase activity | Ldhb, Ldha |
| GO:0005200 | 6 | 60 | 0.006658971 | structural constituent of cytoskeleton | Tnnt2, Tpm1, Dsp, Des, Acta1, Actg1 |
| GO:0003774 | 6 | 60 | 0.006658971 | motor activity | Actc1, Myh6, Myh7, Myl3, Acta1, Mrlcb |
| GO:0005215 | 37 | 873 | 0.006974135 | transporter activity | Hba-a1, Tnni3, Atp2a2, Fabp3, Slc25a4, Mb, Cox6a2, Slc25a3, Cox8h, Cox4i1, Atp5c1, Atp5g3, MGC72942, Atp5e, Atp5a1, Atp5o, Atp5h, Cox6c, Atp5f1, Lpl, Cox5a, Cox5b, Hbb, Cox7b, Vdac3, Uqcrfs1, Uqcrh, Ryr2, Vdac1, Gja1, Uqcrc2, Vdac2, Atp5j, Atp5d, Maoa, Kcnip2, Nedd4a |
| GO:0016453 | 2 | 5 | 0.007515161 | C-acetyltransferase activity | Hadhb, Acat1 |
| GO:0016615 | 2 | 5 | 0.007515161 | malate dehydrogenase activity | Mdh1, Mor1 |
| GO:0004300 | 2 | 5 | 0.007515161 | enoyl-CoA hydratase activity | Hadhb, Ech1 |
| GO:0008430 | 3 | 15 | 0.007875279 | selenium binding | Gpx1, Gpx4, Prdx2 |
| GO:0015662 | 5 | 45 | 0.008374705 | ATPase activity, coupled to transmembrane movement of ions, phosphorylative mechanism | Atp2a2, Atp5g3, MGC72942, Atp5e, Atp5o |
| GO:0051082 | 6 | 64 | 0.009088574 | unfolded protein binding | Cryab, Hspb7, Hspa8, Hspe1, Hspd1, Cd74 |
| GO:0008324 | 19 | 374 | 0.009213589 | cation transporter activity | Atp2a2, Cox6a2, Slc25a3, Cox8h, Cox4i1, Atp5g3, MGC72942, Atp5e, Atp5o, Atp5h, Cox6c, Cox5a, Cox5b, Cox7b, Uqcrfs1, Uqcrh, Ryr2, Uqcrc2, Atp5d |
